# A combination of ethanol and arachidonic acid promotes steatosis and endoplasmic reticulum stress and impairs mitochondrial respiration in H9c2 cardiomyoblasts

**DOI:** 10.1186/s12944-025-02792-3

**Published:** 2025-12-16

**Authors:** Weilun Ai, Emily New, Carol A. Casey, Viswanathan Saraswathi

**Affiliations:** 1 Department of Internal Medicine, Division of Diabetes, Endocrinology, and Metabolism, University of Nebraska Medical Center, Omaha, NE USA; 2 Department of Internal Medicine, Division of Gastroenterology and Hepatology, University of Nebraska Medical Center, Omaha, NE USA; 3 VA Nebraska-Western Iowa Health Care System, Omaha, NE USA

**Keywords:** Arachidonic acid, Ethanol, Cardiomyopathies, H9c2 cells

## Abstract

Arachidonic acid (AA), an omega-6 polyunsaturated fatty acid, is abundant in animal-derived food and is widely present in phospholipids of plasma membrane. Recent studies reported that ethanol exposure leads to the activation of prostaglandin signaling via increasing the levels of AA and its metabolites in cardiomyocytes. To test the hypothesis that AA contributes at least in part, to ethanol-induced cardiomyocyte injury, a chronic ethanol feeding model was used, in which male Wistar rats were fed Lieber-Decarli ethanol diet 6.7% (v/v) or isocaloric control diet for 6 weeks. Gas chromatography analysis indicated that ethanol exposure increased the AA content in rat myocardial phospholipids along with increased protein levels of endoplasmic reticulum (ER) stress markers and a decrease in the level of NADH: ubiquinone oxidoreductase subunit B8, a mitochondrial complex I subunit. In addition, an in vitro model was used in which H9c2 cells, a rat cardiomyoblast cell line, were exposed to AA and/or ethanol (ET), and markers of steatosis and endoplasmic reticulum stress, and mitochondrial respiration were assessed. Of note, AA supplementation potentiated ethanol-induced steatosis. H9c2 cells receiving ET + AA showed an increase in the expression of ER stress markers, including glucose-regulated protein 78 and activating transcription factor 4, compared with controls. Interestingly, compared to ET treatment, ET + AA treatment led to a significant decrease in basal respiration and ATP-linked respiration, indicating an impaired mitochondrial respiration in H9c2 cardiomyoblasts. Finally, inhibiting long-chain acyl CoA synthases by Triacsin C attenuated ET + AA treatment-induced steatosis but increased mitochondrial respiration in H9c2 cells. Collectively, these data suggested that AA supplementation promotes ethanol-induced steatosis and endoplasmic reticulum stress with a concomitant impairment in mitochondrial respiration in H9c2 cardiomyoblasts, and Triacsin C treatment inhibits steatosis but enhances mitochondrial respiration possibly via altered fatty acid partitioning between synthetic and oxidative processes.

**Supplementary Information:**

The online version contains supplementary material available at 10.1186/s12944-025-02792-3.

## Introduction

Arachidonic acid (AA), an omega-6 polyunsaturated fatty acid (FA), is abundant in animal-derived food [1]. AA is widely present in phospholipids of the cell plasma membrane and mediates diverse physiological processes. Currently, AA is gaining increased attention because AA exerts significant effects on human health and disease. Previous studies in human subjects have reported that supplementation of AA exerts beneficial effects on muscle function [2, 3]. Moreover, dietary supplementation of AA has been shown to improve brain damage-induced cognitive dysfunction [4]. In cardiovascular disease (CVD) studies, AA has shown protective effects on attenuating high fat diet-induced inflammation in the heart [5]. A recent in vivo study supports a beneficial role of AA in ameliorating diabetes mellitus-induced cardiomyopathy [6]. However, other studies suggest the role for AA and AA signaling pathways in mediating adverse cardiovascular effects [7, 8]. For example, a Mendelian randomization study showed a positive correlation of circulating AA levels with the risk of atherosclerotic cardiovascular diseases [9]. Moreover, free AA is derived from the plasma membrane during cellular stress and is enzymatically converted into lipid mediators by tissue-specific synthases, leading to AA cytotoxicity. Specifically, bioactive lipid metabolites from AA metabolism promote inflammation, oxidative stress, hypertrophy, and endoplasmic reticulum (ER) stress, thereby contributing to different CVD [10]. In addition, previous research has documented increased oxidative stress and derangement of calcium handling in the neonatal rat cardiomyocytes exposed to AA [11, 12].

Heavy alcohol use induces alcohol-associated cardiomyopathy (ACM), which accounts for 6.3% of all cardiomyopathy-associated mortality worldwide [13]. ACM is characterized by impaired myocardial contractility and dilation of ventricles [14]. Several factors are responsible for the development of ACM, including oxidative stress, dysregulated lipid metabolism, mitochondrial dysfunction, and defects in energy production [14]. Notably, ethanol consumption increases triglyceride deposition in rat myocardium along with impaired mitochondrial dysfunction, which, in turn, results in impaired ATP production and compromised cardiac contractile function [15]. Interestingly, a recent study using human induced pluripotent stem cell-derived cardiomyocytes reported that long-term ethanol exposure leads to the activation of prostaglandin signaling by increasing the levels of AA and AA-derived metabolites, which are associated with impairment of mitochondrial respiration [16]. Another recent study indicates that mice fed an ethanol diet show activation of thromboxane A2 signaling pathway, a member of AA signaling pathway, with concomitant increases in inflammation and collagen deposition in the mouse myocardium [17].

Existing studies suggest that animal models using ethanol alone are not able to recapitulate severe organ injuries found in humans, and ethanol-induced organ injury is exacerbated by a second insult [18]. Of note, a high-fat diet, enriched with polyunsaturated FA, potentiates ethanol-induced steatosis, inflammation, and fibrosis [18]. Currently, it is estimated that AA intake is increased in humans from both developed and developing countries [3]. Studies have reported the detrimental effects of AA on alcohol-related organ injuries. For example, dietary supplementation of AA has been documented to aggravate ethanol-induced hepatic steatosis and oxidative stress in rats [19]. However, the role and mechanisms by which a combination of AA and ethanol alters cardiomyocyte injury remain elusive. Considering the high intake of AA and alcohol among people in Western countries [20, 21], it is crucial to investigate whether AA aggravates alcohol-induced myocardial lesions. The central hypothesis is that a combination of AA and ethanol elicits cardiomyocyte injury via reciprocally regulating steatosis and mitochondrial respiration. To test the hypothesis, an in vitro model was utilized, in which H9c2 cells, a rat cardiomyoblast cell line, were exposed to AA in the presence or absence of ethanol.

## Materials and methods

### Reagents

The reagents, chemicals, and supplies used in the current study includes: Dulbecco’s Modified Eagle’s Medium (DMEM, SH30243.01, Cytiva, MA, USA), phenol red-free DMEM (SH30284.01, Cytiva), XF DMEM medium (pH 7.4, 103575-100, Agilent Technologies, CA, USA), AA (U-71 A, Nu-Chek-Prep, Inc., MN, USA), ethanol (111000200, Greenfield Global, Canada), Triacsin C (10007448, Cayman Chemical, MI, USA), oil-red O powder (O0625, Sigma-Aldrich, MO, USA), Ponceau S solution (59803, Cell Signaling Technology, MA, USA), PrestoBlue^®^ Cell Viability Reagent (Thermo Fisher Scientific, MA, USA), hydrogen peroxide assay kit (ab102500, Abcam, Cambridge, UK), TRIzol reagent (Ambion Life Technologies, MA, USA), NuGEN Universal Plus mRNA-Seq library kit (TECAN, CA, USA), 5× iScript reverse transcription supermix (1708841, Bio-Rad, CA, USA), iQ supermix (1708862, Bio-Rad), oligomycin (O4876, Sigma-Aldrich, MO, USA), carbonyl cyanide 4-(trifluoromethoxy) phenylhydrazone (FCCP, C2920, Sigma-Aldrich), rotenone (R8875, Sigma-Aldrich), antimycin A (A8674, Sigma-Aldrich), Taqman™ real-time PCR primers (Applied Biosystems, MA, USA), including *Atf4* (Rn00824644_g1), *Ddit* (Rn00492098_g1), *18s* (Applied Biosystems, 4352930E), antibodies against 4-Hydroxynonenal (4-HNE, ab46545, Abcam), glucose-regulated protein 78 (GRP78, 11587-1-AP, Proteintech, IL, USA), aldehyde dehydrogenases 1/2 (ALDH1/2, sc-166362, Santa Cruz Biotechnology, TX, USA), activating transcription factor 4 (ATF4, 10835-1-AP, Proteintech), C/EBP homologous protein (CHOP, 15204-1-AP, Proteintech), oxidative phosphorylation (OXPHOS) rodent antibody (45–8099, Thermo Fisher Scientific), anti-rabbit secondary antibody conjugated to horseradish peroxidase (7074, Cell Signaling Technology), anti-mouse StarBright™ Blue 520 fluorescence secondary antibody (12005867, Bio-Rad), enhanced chemiluminescence reagent (1705062, Bio-Rad).

### Cell culture model

H9c2 cell (CRL-1446™), a rat cardio-myoblast cell line, was purchased from ATCC (VA, USA). Cells were cultured in DMEM containing 10% fetal bovine serum (FBS). H9c2 cardiomyoblasts were exposed to 100 mM ethanol with/without 50 µM AA in the free fatty acid form for 24 h. The alcohol concentration at 100 mM was utilized to induce cardiomyocyte injury in vitro following the previous studies [22]. The concentration at 50 µM AA was used for the treatment as described previously [11]. Treatment with AA was conducted using DMEM supplemented with 10% FBS as the source of albumin [23, 24]. AA was solubilized in ethanol and subsequently added to DMEM with 10% FBS prior to treating H9c2 cardiomyoblasts. Cells were divided into four groups: Control, AA, ET, and ET + AA. To inhibit long-chain fatty acyl-CoA synthetase (ACSL) activity, cells were treated with 2 µM Triacsin C for 24 h [25]. Cells were divided into two groups: ET + AA and ET + AA + Triacsin C.

### Animal model

Male Wistar rats (175 to 200 g) were purchased from Charles River Labs (Portage, MI, USA). Rats were randomized into two groups. Rats were weight-matched and fed 6.7% (v/v) ethanol-containing Lieber–DeCarli diets (ET, Dyets Inc., PA, USA, *n* = 7) or isocaloric control diet (CON, Dyets Inc., PA, USA, *n* = 8) for 6 weeks after a four-day ramp-up period from 0% to 6.7% ethanol diet to induce cardiac dysfunction as described previously [22, 26]. The intakes of the ethanol-containing diet and the control diet were recorded daily. Next, rats were euthanized using isoflurane anesthesia at which time blood was collected from the vena cava, and hearts. Protocols involved in in vivo experiments were approved by the Institutional Animal Care and Use Committee at the VA Nebraska-Western Iowa Health Care System Research Service.

### Oil-red O staining

H9c2 cells (1 × 10^4^ cells/well) were seeded in chamber slides. After treatment, cardiomyoblasts were fixed using 4% paraformaldehyde solution. Next, an oil-red O stock solution was prepared by dissolving 0.5 g of oil-red O powder in 100 mL of absolute isopropanol. Later, 30 mL of oil-red O solution was mixed with 20 mL of distilled water, and the mixture was filtered to make oil-red O assay solution. H9c2 cardiomyoblasts were incubated in the assay solution for 20 min after washing by 1× PBS twice. Excessive staining was removed through washing cardiomyoblasts with 40% isopropanol twice. Finally, cells were counterstained with hematoxylin and visualized for quantification [27].

### Cell viability assay

H9c2 cells (1 × 10^4^ cells/well) were seeded in the 96-well plate containing 90 µL per well of culture medium. After treatments, PrestoBlue^®^ Cell Viability Reagent (10 µL per well) was added, and cells were placed in a cell culture incubator for 30 min. Then, the fluorescence was read by SpectraMax plate reader at 560/590 nm.

### RNA sequencing

Total RNAs from H9c2 cells were extracted using the TRIzol reagent. The RNA integrity was assessed first, and RNA-sequencing was performed on the Illumina NextSeq 550 system. RNA sequencing was performed using 500 ng of total RNA from each sample using the NuGEN Universal Plus mRNA-Seq library kit following the manufacturers instruction. The raw data (FASTQ files) was submitted in Gene Expression Omnibus database (GSE292883). Raw reads were processed to yield Fragments Per Kilobase of transcript per Million mapped reads and the expression value of each gene was further normalized. Genes with an adjusted *P* < 0.05 were considered as significantly altered. Based on those significantly altered genes, ingenuity pathway analysis (IPA) was carried out to explore canonical pathways altered due to ET + AA treatment.

### Real-time PCR

RNA from H9c2 cell was reverse-transcribed utilizing iScript supermix. Real-time PCR was further performed to measure the transcript levels of *Atf4* and *Ddit*. The Taqman™ real-time PCR primer probes from Applied Biosystems, were used to detect the mRNA levels of genes, including *Atf4* and *Ddit*. Transcript levels were normalized to *18s*. A ΔΔCT method was utilized for the quantification.

### Western blotting

H9c2 cells were homogenized in RIPA lysis buffer containing phosphatase-proteinase inhibitor cocktail. Samples were centrifuged at 12,000 × *g* for 10 min to collect the supernatants. Next, proteins were separated by electrophoresis using SDS polyacrylamide gels and immunoblotted to polyvinylidene difluoride membranes. After the transfer, the membranes were incubated in Ponceau S solution for two minutes and rinsed with distilled water. Furthermore, Ponceau S staining was visualized by the ChemiDoc System (Bio-Rad). To remove Ponceau S staining, membranes were washed with TBS buffer containing 0.5% Tween-20 (TBST) three times. Then, membranes were blocked with 5% bovine serum albumin for 1 h. After washing three times with TBST, membranes were incubated with primary antibodies overnight at 4 °C. Later, membranes were washed and subsequently incubated with anti-rabbit secondary antibody conjugated to horseradish peroxidase or anti-mouse StarBright™ Blue 520 fluorescence secondary antibody at a 1:5000 dilution for 1 h at room temperature. Secondary antibody signals were revealed by enhanced chemiluminescence reagent. Protein bands were visualized using the ChemiDoc System (Bio-Rad). Ponceau S staining and protein bands were quantified using ImageJ. The total protein level was determined by Ponceau S staining, which was used for western blot normalization.

### Hydrogen peroxide assay

H9c2 cells (2 × 10^6^ cells/flask) were seeded in T-25 flask containing 3 ml of phenol red-free DMEM with 10% FBS. After treatment, media were collected, and media hydrogen peroxide (H_2_O_2_) levels were measured utilizing the H_2_O_2_ assay kit. After color development, the plate was read at 570 nm. Next, H9c2 cells were collected and digested by RIPA buffer. The total amount of protein in each flask was measured. The protein levels were used to normalize H_2_O_2_ concentration.

### Seahorse mitochondria stress test

H9c2 cells (5 × 10^4^ cells/well) were seeded in the XF24 cell culture microplate (Agilent Technologies, CA). The next day, H9c2 cardiomyoblasts were treated with ET and/or AA as described above. After treatment, XF sensor was hydrated with calibrant solution at 37 °C overnight. At the time of Seahorse mitochondria stress test, the assay medium was prepared using the XF DMEM medium (pH 7.4) containing 2.5 mM glucose and 1 mM pyruvate. The assay medium was kept at 37 °C in a non-CO_2_ incubator. Then, the medium in each well was replaced by 525 µL of assay medium and 75 µL of injection compounds, including oligomycin, FCCP, and a rotenone-antimycin A mixture. Those reagents were loaded through the corresponding injection ports, leading to the final concentrations of all compounds at 1 µM. The oxygen consumption rate (OCR) was recorded by the Seahorse XFe24 Analyzer (Agilent Technologies). Basal and maximal respiration, ATP-associated respiration, and coupling efficiency were calculated following the equations from Agilent Technologies: (1) basal respiration = (the last measurement of OCR before first injection) – (non-mitochondrial respiration rate); (2) maximal respiration = (the measurement of maximum OCR after FCCP injection) – (non-mitochondrial respiration); (3) ATP-linked respiration = (the last measurement of OCR before oligomycin injection) – (the measurement of minimum rate after the oligomycin injection); (4) coupling efficiency = (ATP-linked respiration)/(basal respiration) × 100.

### Gas chromatography (GC) analysis

Rat left ventricle samples were used to measure myocardial phospholipid (PL) levels and PL composition. PL were measured using the GC analysis [28]. Briefly, lipid extraction was performed using the method established by Folch-Lees [29]. Next, individual lipid classes were separated by thin-layer chromatography using the Silica Gel 60 A plates and visualized by rhodamine 6G. Phospholipids were scraped from the plates and methylated using BF3/methanol. The methylated fatty acids were collected and further analyzed by GC using an HP 5890 gas chromatograph.

### Statistics

All data are shown as mean ± standard deviation (SD) and analyzed by a one-way ANOVA (Newman-Keuls multiple comparison test). Single factor comparisons were performed by Student’s *t*-test. Pearson correlation was utilized to ascertain the association of AA content in phospholipids with markers of ER stress and mitochondrial respiration in rat myocardium. GraphPad Prism 10.1 was used for the statistical analysis. Differences at *P* < 0.05 were considered statistically significant.

## Results

### Effects of ethanol and/or AA on steatosis in H9c2 cardiomyoblasts

**Fig. 1:**
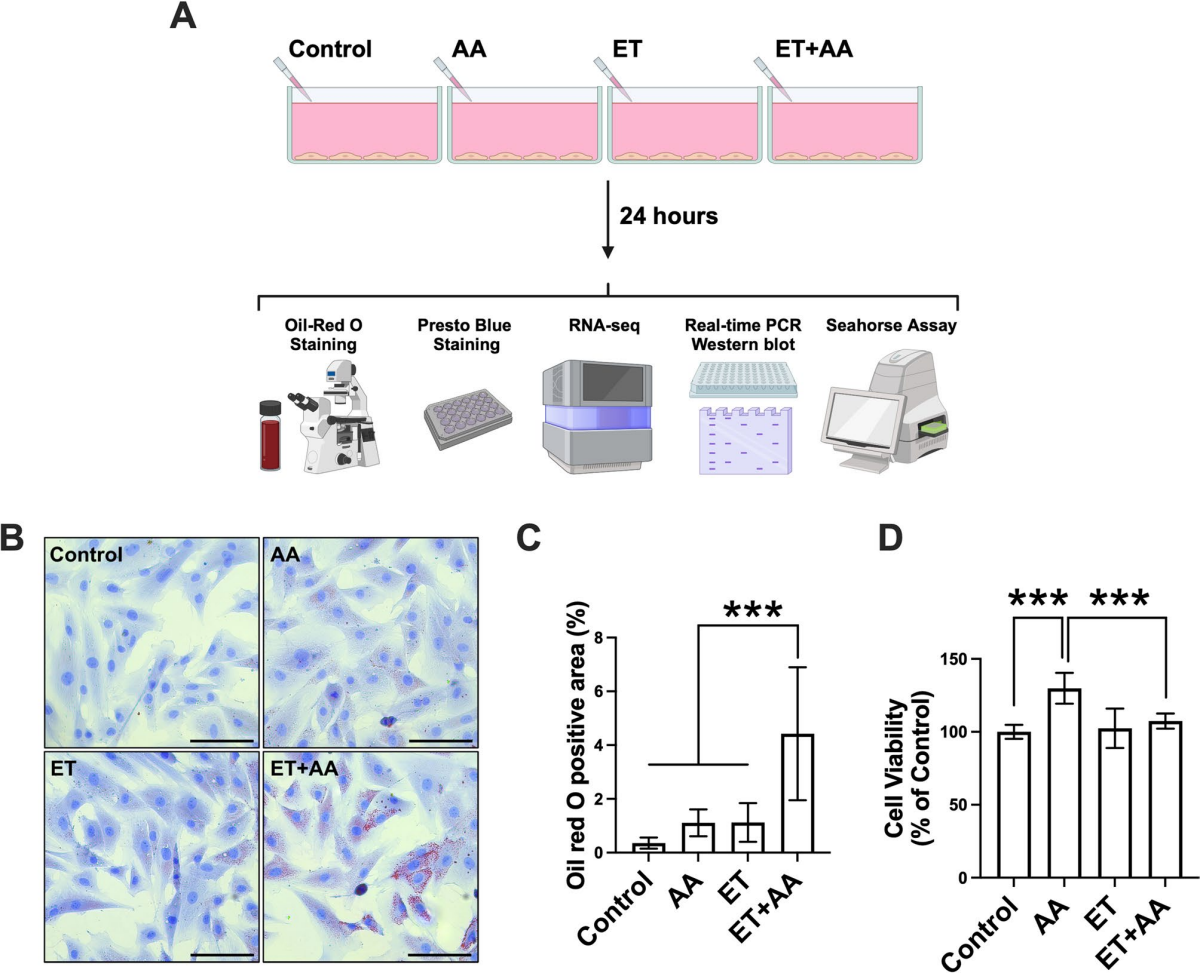
The effects of ethanol and/or AA on steatosis and viability of H9c2 cardiomyoblasts. **A** Schematic overview for in vitro experimental design (generated by BioRender). **B** Representative pictures of oil-red O staining of H9c2 cells, scale bar = 100 μm. **C** Quantification of oil-red O staining, *n* = 6 per group. **D** Cell viability as evaluated by PrestoBlue assay, *n* = 12 wells/group. Values are presented as mean ± SD, ***P < 0.001

To ascertain the effects of AA in altering alcohol-induced early cardiomyocyte injury, H9c2 cells received 100 mM ethanol treatment with/without AA supplementation at 50 µM for 24 h (Fig. 1A). Toraason et al.. used 50 µM of AA and demonstrated that AA promotes H_2_O_2_-induced oxidative stress in neonatal cardiomyocytes [11]. In addition, Haworth et al. reported that AA at 50 µM concentration induced mitochondrial depolarization in isolated rat cardiomyocytes [30]. The present study shows a mild increase in lipid accumulation in H9c2 cells exposed to either AA or ET compared with controls (Fig. 1B and C). Interestingly, H9c2 cells exposed to ET + AA showed an evident increase in lipid deposition compared to all other groups (*P* < 0.001, Fig. 1B and C). Next, cell viability was determined to test if ethanol and/or AA altered the viability of H9c2 cells. Interestingly, AA supplementation increased cell numbers compared to controls (Fig. 1D). Nevertheless, ET + AA-treated cells showed a reduction in cell numbers compared to the AA group, which suggests that ethanol abolished the effect of AA in increasing cell numbers (Fig. 1D).

### Effects of ethanol and/or AA on oxidative stress in H9c2 cells

**Fig. 2:**
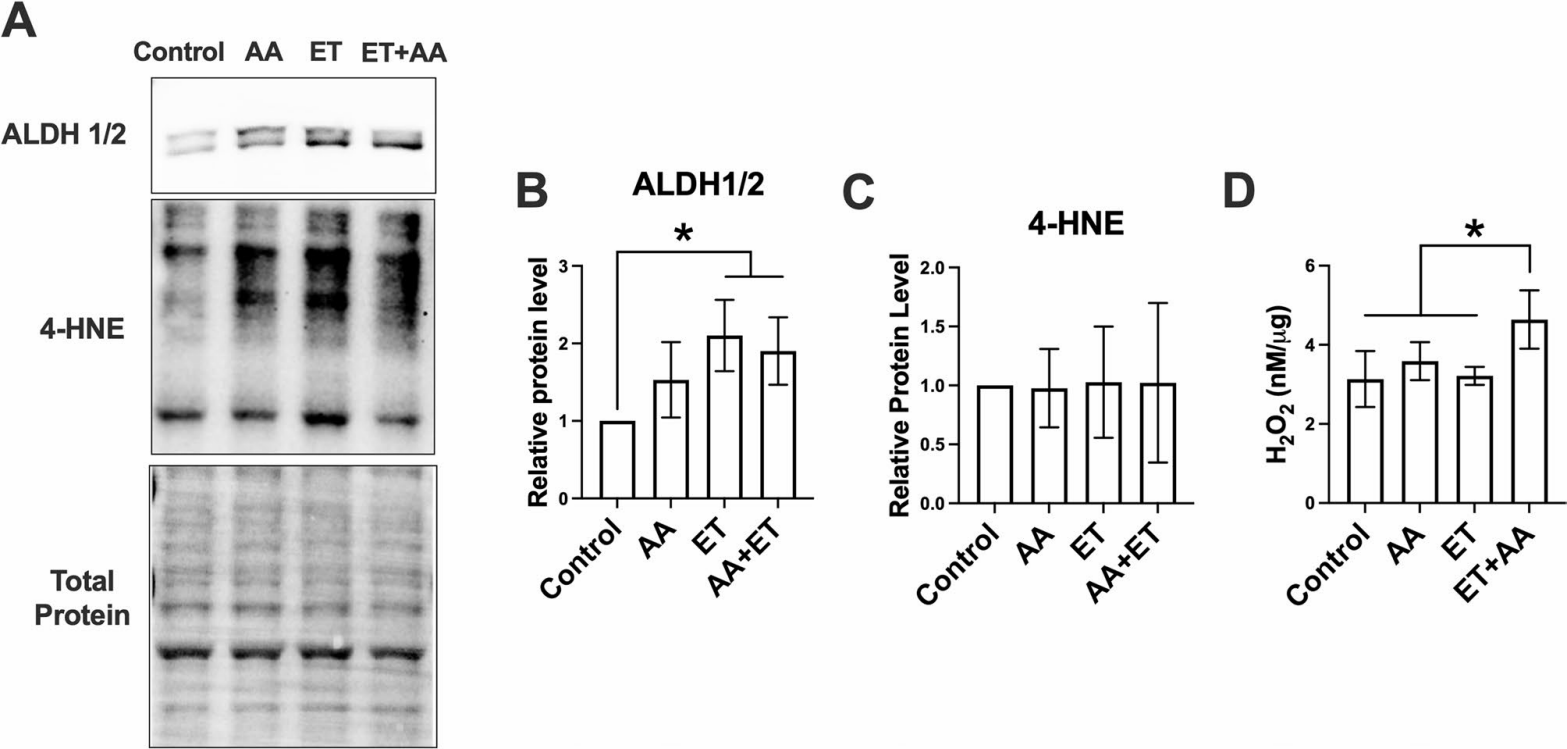
The effects of ethanol and/or AA on oxidative stress in H9c2 cardiomyoblasts. **A** Western blot images for ALDH1/2 and 4-HNE in H9c2 cell samples. **B**&**C** Densitometric analysis of the band intensities for ALDH1/2 and 4-HNE. Values are normalized to total protein. **D** H_2_O_2_ level in culture media. *n* = 4 per group. Values are expressed as mean ± SD. *P < 0.05

Ethanol induces oxidative stress in cardiomyocytes which were associated with cardiac steatosis [14]. To examine whether AA supplementation enhances these adverse effects of ethanol, markers of oxidative stress were analyzed. Interestingly, H9c2 cardiomyoblasts exposed to ET had an increased protein level of ALDH1/2 (*P* < 0.05, Fig. 2A and B), an enzyme responsible for acetaldehyde clearance and preventing protein adduct formation, which indicates a feedback regulation upon ethanol exposure. However, H9c2 cardiomyoblasts exposed to ethanol and/or AA showed no alteration in the level of 4-HNE, an aldehyde product of lipid peroxidation, indicating that despite an increase in ALDH1/2, there is no reduction in lipid peroxidation (Fig. 2A and C). Next, the concentration of H_2_O_2,_ an oxidizing agent, was measured in the culture media from cardiomyoblasts challenged with ET and/or AA. ET + AA-treated H9c2 cardiomyoblasts showed a small but significant increase in H_2_O_2_ content in media compared with all groups (*P* < 0.05, Fig. 2D), indicating that ET + AA promotes oxidative stress in cardiomyoblasts.

### Roles of ethanol and/or AA on markers of ER stress in H9c2 cardiomyoblasts

**Fig. 3:**
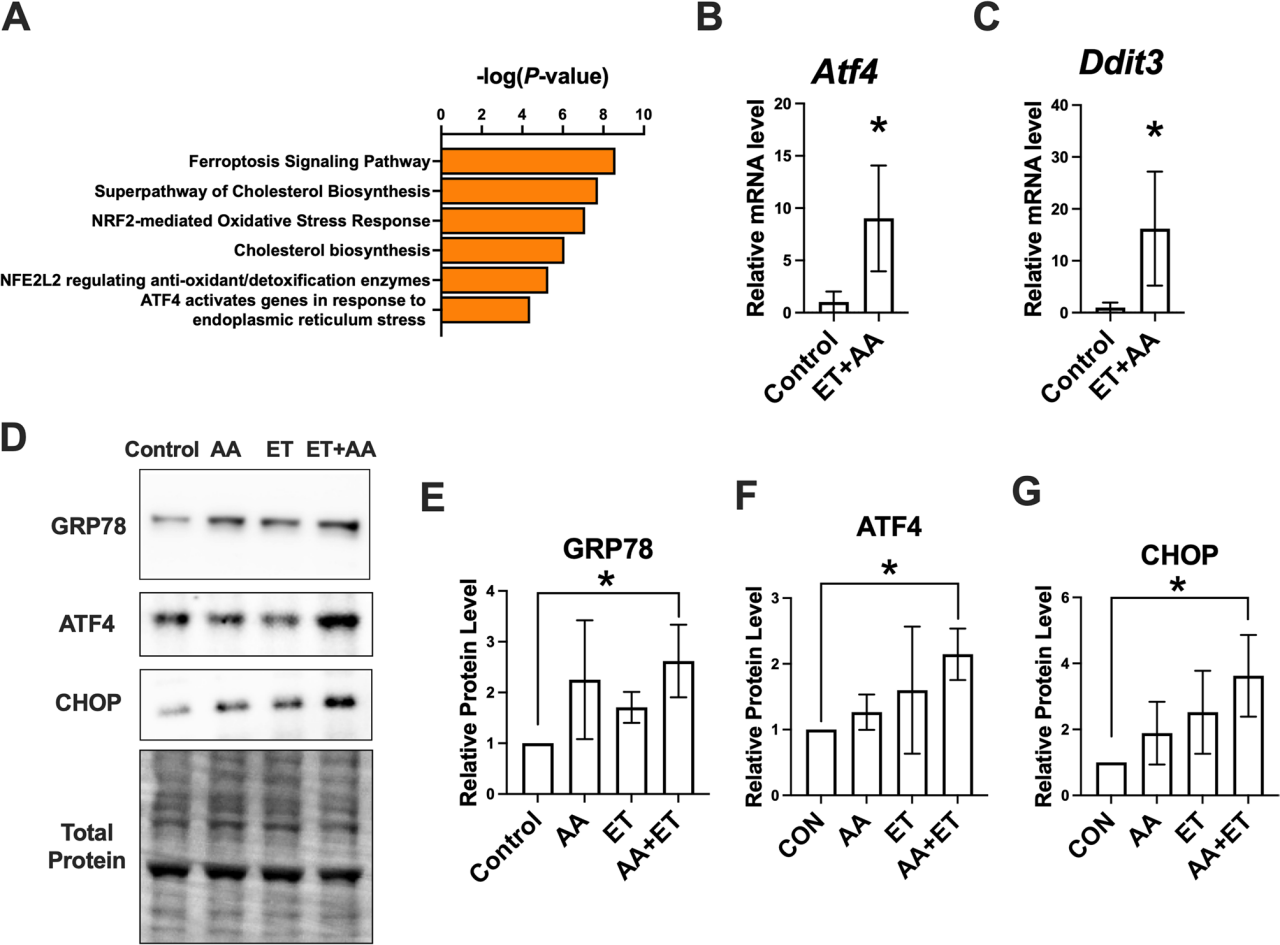
The effects of ethanol and/or AA on ER stress in H9c2 cardiomyoblasts. **A** Upregulated signaling pathways in H9c2 cardiomyoblasts in response to ET + AA treatment as determined via RNA-seq. **B**&**C** Real-time PCR analysis showing transcript levels for *Atf4* and *Ddit3* of H9c2 cells. **D** Western blot images for GRP78, ATF4, and CHOP. **E**-**G** Densitometric analysis of the band intensities for GRP78, ATF4, and CHOP, values are normalized to total protein, *n* = 4 per group. Values are reported as mean ± SD. *P < 0.05

To assess the effects of ET + AA in the overall changes in gene expression in H9c2 cells, an RNA-sequencing analysis in control and ET + AA-treated cells was performed, which revealed that 1211 genes were significantly altered in H9c2 cardiomyoblasts exposed to ET + AA versus controls (Suppl. Table 1). IPA was carried out to investigate altered signaling pathways upon ET + AA treatment. Figure 3A shows the selected upregulated signaling pathways involved in various cellular processes, including ER stress and ferroptosis. Regarding ER stress-related genes, ET + AA-treated cells showed an increase in *Atf4* and *Ddit3*, a gene encoding CHOP. In line with the RNA-seq data, real-time PCR analysis further confirmed that ET + AA exposure increased transcript levels of *Atf4* and *Ddit3* in cardiomyoblasts (*P* < 0.05, Fig. 3B and C). Regarding the protein levels of ER stress markers, H9c2 cardiomyoblasts receiving ET + AA had a notable increase in the protein level of GRP78 (*P* < 0.05), an important ER stress regulator, compared with controls (Fig. 3D and E). ET + AA-treated cells also showed increased protein levels of ATF4 and CHOP, compared to controls (Fig. 3D, F and G). Collectively, AA supplementation potentiated ethanol-induced ER stress in H9c2 cells.

### Effects of ethanol and/or AA on mitochondrial respiration in H9c2 cardiomyoblasts

**Fig. 4:**
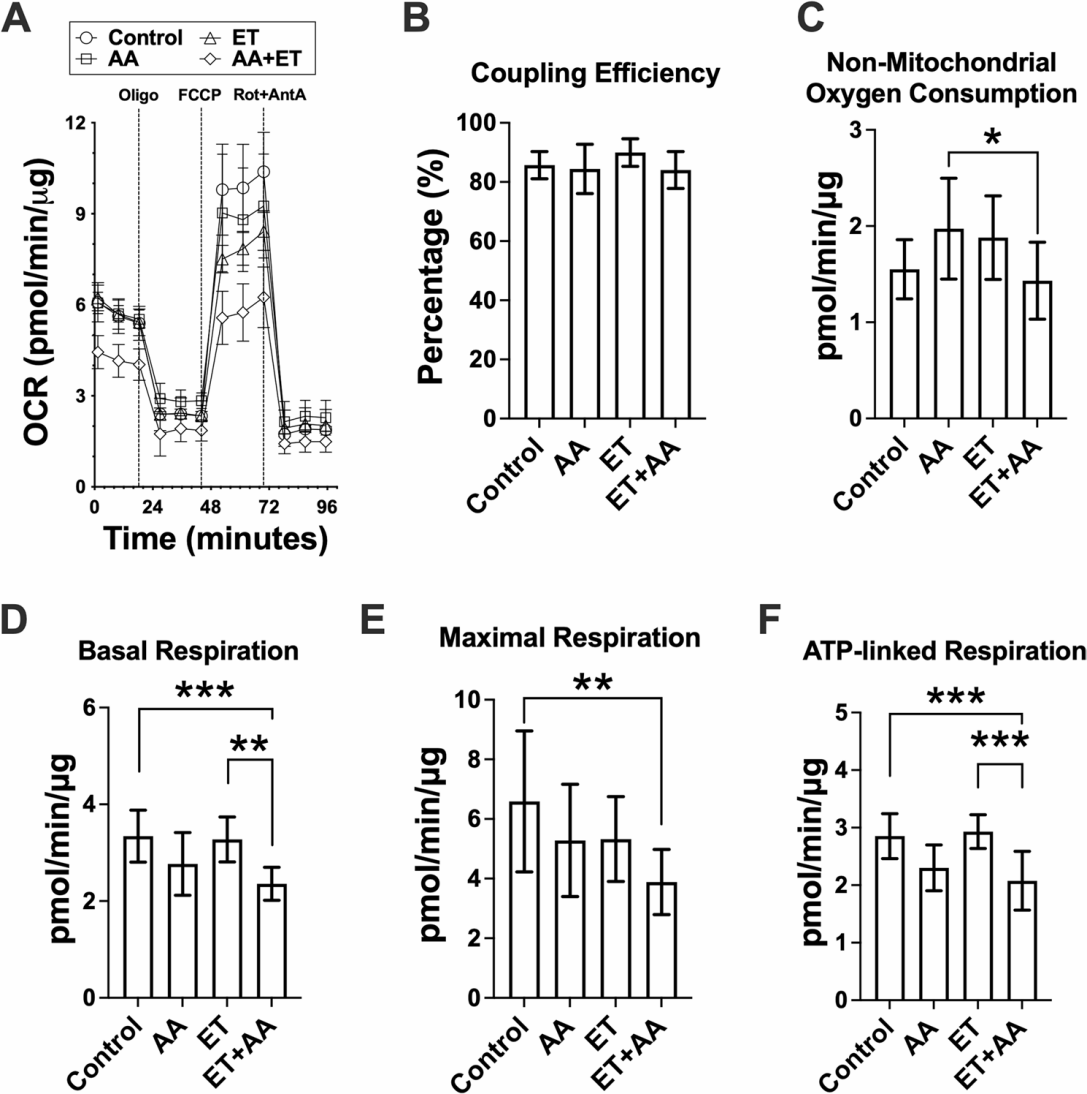
The effects of ethanol and/or AA on mitochondrial respiration in H9c2 cardiomyoblasts. **A** Representative picture tracing oxygen consumption rate (OCR) in H9c2 cells following sequential addition of oligomycin, FCCP, and rotenone/antimycin A. **B**-**F** Bar charts show the quantification of coupling efficiency, non-mitochondrial respiration, basal respiration, maximal respiration, and ATP-linked respiration. *n* = 9–10 wells per group. Values are expressed as mean ± SD, *P < 0.05, **P < 0.01, ***P < 0.001

There is a strong link between AA and mitochondrial dysfunction [31]. The mitochondrial stress assay was performed to test whether AA in the presence of ethanol leads to impairments in mitochondrial respiration. Seahorse flux analysis was conducted to assess mitochondrial respiration in response to stress. Alterations in oxygen consumption rate (OCR) of each well were traced, followed by the sequential treatment of oligomycin, FCCP, and rotenone + antimycin A, as shown in Fig. 4A. AA supplementation had no effect on coupling efficiency and non-mitochondrial oxygen consumption of H9c2 cardiomyocytes compared with controls (Fig. 4B and C). Of note, ET + AA-treated cells showed a significant reduction in non-mitochondrial oxygen consumption (*P* < 0.05) compared with cardiomyoblasts exposed to AA alone (Fig. 4C). In addition, ET or AA treatment had no significant effects on the basal respiration, maximal respiration, and ATP-linked respiration compared with controls (Fig. 4D-F**)**. However, a mixture of AA and ethanol led to an evident decrease in basal respiration (2.357 ± 0.3394 vs. 3.342 ± 0.5376, *P* < 0.001, Fig. 4D), maximal respiration (3.887 ± 1.094 vs. 6.592 ± 2.364, *P* < 0.01, Fig. 4E), and ATP-linked respiration (2.077 ± 0.5119 vs. 2.852 ± 0.3908, *P* < 0.001, Fig. 4F) compared with controls. Furthermore, ET + AA treatment led to a substantial decrease in basal respiration and ATP-linked respiration compared to treatment with ethanol alone. These results indicate that a combination of AA and ethanol attenuates mitochondrial respiration in H9c2 cells.

### Effects of ethanol and/or AA on the expression of proteins related to oxidative phosphorylation in H9c2 cells

**Fig. 5:**
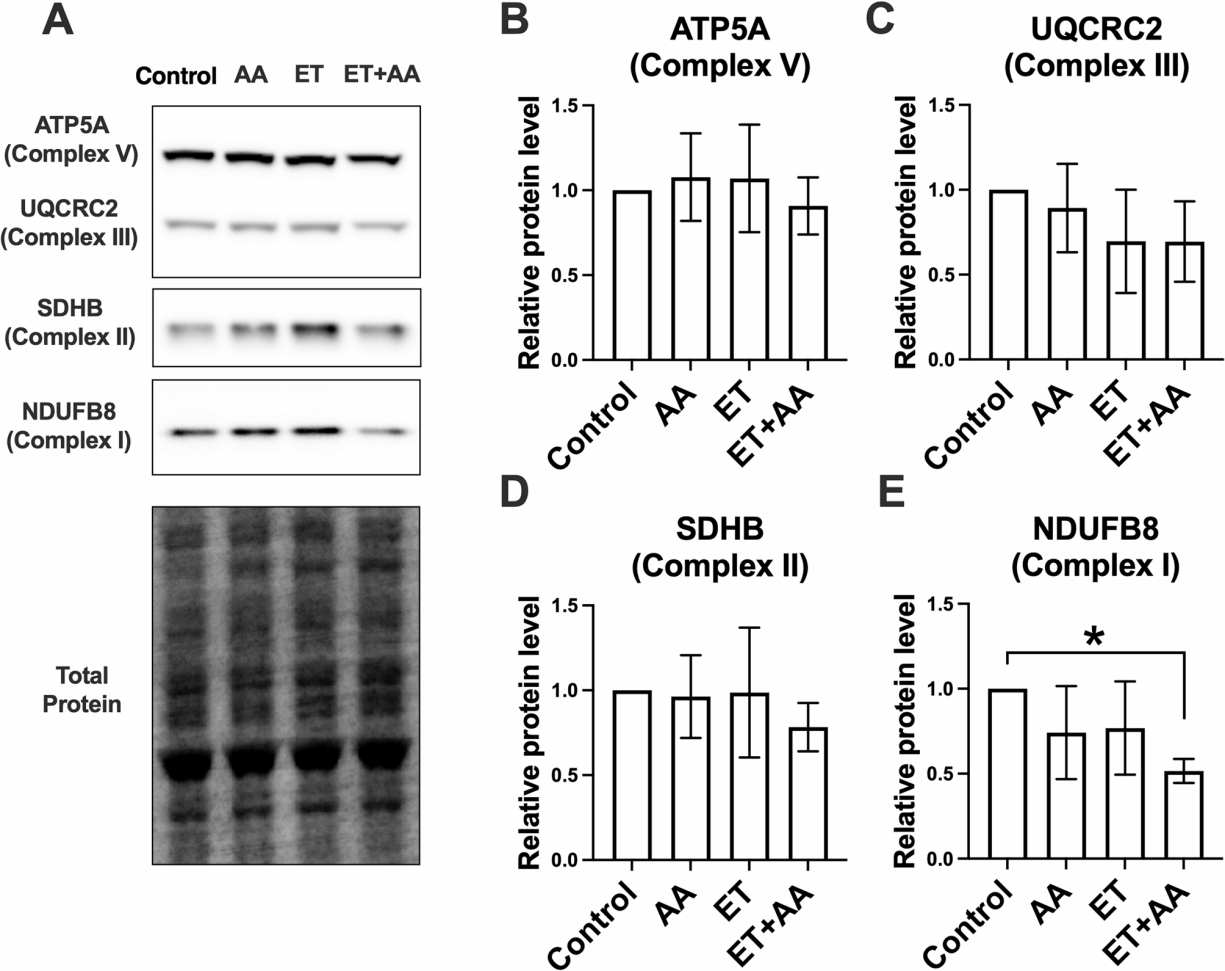
The effects of ethanol and/or AA on proteins involved in OXPHOS in H9c2 cardiomyoblasts. **A** Western blot images for ATP5A, UQCRC2, SDHB, and NDUFB8 in H9c2 cell samples. **B**-**E** Densitometric analysis of the band intensities for ATP5A, UQCRC2, SDHB, and NDUFB8. Values are normalized to total protein, *n* = 4 per group. Values are expressed as mean ± SD. *P < 0.05

Oxidative phosphorylation (OXPHOS) system contains five complexes and is the key component of mitochondria for the generation of ATP. As mentioned, AA in the presence of ethanol decreases basal, maximal, and ATP-linked respiration in H9c2 cells. Therefore, the expression of proteins related to OXPHOS in mitochondria was measured (Fig. 5A). The results showed that H9c2 cells exposed to ET + AA did not alter the protein level of ATP synthase F1 subunit α (ATP5A, Fig. 5B). Additionally, ET + AA-treated cells exhibited a trend towards a decreased protein level of ubiquinol-cytochrome C reductase core protein 2 (UQCRC2, Fig. 5C), and succinate dehydrogenase complex iron sulfur subunit B (SDHB, Fig. 5D) compared with control cells. Of note, the ET + AA group showed a significant decrease in the protein level of NADH: ubiquinone oxidoreductase subunit B8 (NDUFB8), a component of mitochondrial complex 1 (*P* < 0.05, Fig. 5E). Collectively, these data indicate that a combination of AA and ethanol leads to an impairment in mitochondrial respiration, specifically through down-regulation of mitochondrial complex I component.

### Effects of ethanol on FA composition of rat myocardial phospholipids

**Fig. 6:**
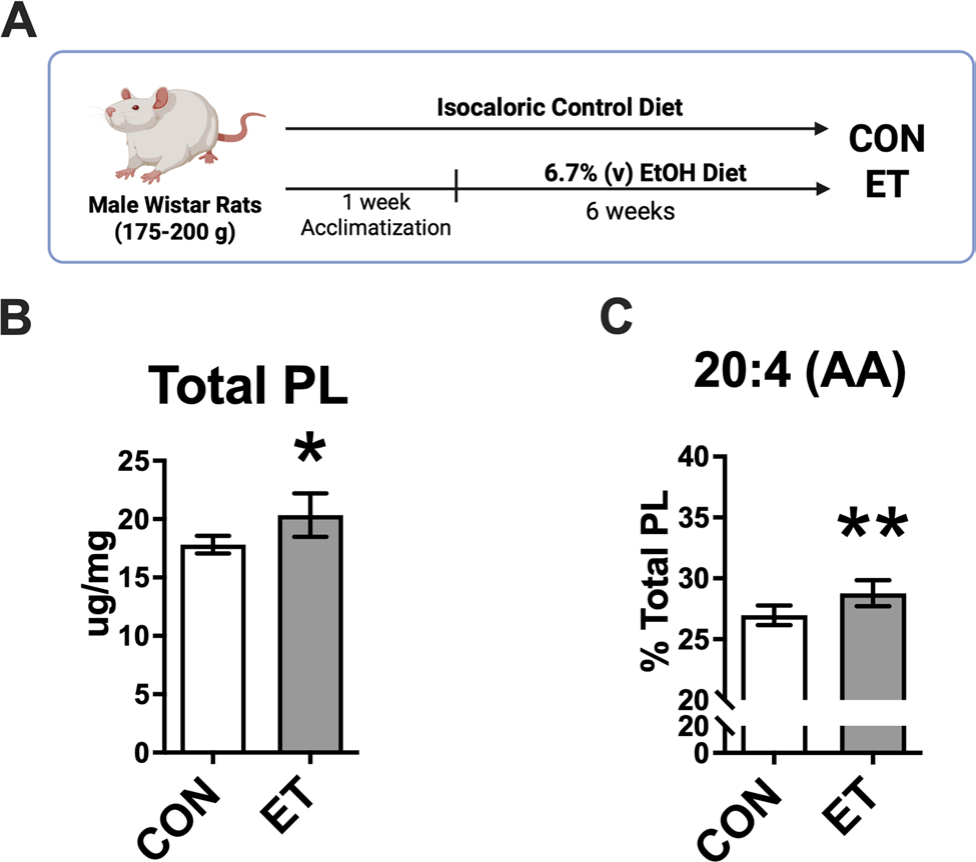
The effects of chronic ethanol consumption on fatty acid composition in myocardial phospholipids. **A** Schematic overview of the in vivo experimental design (created with BioRender). **B** The level of total myocardial phospholipids determined by gas chromatography. **C** The level of AA in myocardial phospholipids, *n* = 6 per group. Values are expressed as mean ± SD, *P < 0.05, **P < 0.01

Rats exposed to six-week chronic ethanol feeding (Fig. 6A) showed an increased total myocardial phospholipid level compared with control rats (*P* < 0.05, Fig. 6B). Moreover, chronic ethanol consumption increased AA level in phospholipids of rat myocardium (*P* < 0.01, Fig. 6C), indicating that ethanol promotes AA partitioning to phospholipids.

### Effects of chronic ethanol diet on markers of ER stress and OXPHOS in the rat myocardium

**Fig. 7:**
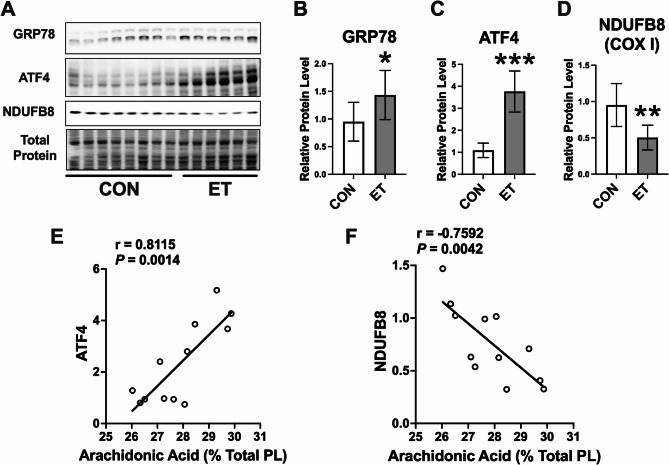
The effects of chronic ethanol on proteins involved in ER stress and OXPHOS in rat myocardium. **A** Western blot images for GRP78, ATF4, and NDUFB8 in rat myocardium. **B**-**D** Densitometric analysis of band intensities for GRP78, ATF4, and NDUFB8. Values are normalized to total protein, *n* = 6–8 per group. Values are expressed as mean ± SD. *P < 0.05, **P < 0.01, ***P < 0.001. **E**&**F** Pearson correlation plots showing the correlation of myocardial AA phospholipid level with the protein levels of ATF4 or NDUFB8, *n* = 6 per group

Six-week ethanol feeding model as described above was used to ascertain whether ethanol induces ER stress and compromises OXPHOS of rat myocardium. ET-fed rats showed an increase in myocardial protein levels of GRP78 (*P* < 0.05, Fig. 7A and B) and ATF4 (*P* < 0.001, Fig. 7A and C), indicating an increase in ER stress. In addition, ethanol decreased NDUFB8 expression in rat myocardium (*P* < 0.01, Fig. 7A and D), suggesting that OXPHOS is also impaired in these rats. AA-phospholipids exert a pivotal role in regulating mitochondrial function and ER stress [32, 33]. As mentioned earlier, rats exposed to a chronic ethanol diet exhibited increased AA levels in myocardial phospholipids (Fig. 6C). Next, to examine whether the increase in AA content in phospholipids is associated with markers of ER stress and mitochondrial respiration, Pearson correlation analysis was performed which showed that the AA level in myocardial phospholipids is positively correlated with ATF4 expression (Fig. 7E) and AA exhibited a negative correlation with NDUFB8 expression (Fig. 7F). Collectively, these data suggest that ethanol increases AA level in myocardial phospholipids, which exhibits a positive and negative correlation, respectively, with markers of ER stress and mitochondrial respiration.

### Effect of inhibiting ACSL activity on steatosis and mitochondrial respiration in H9c2 cells exposed to AA and ethanol

**Fig. 8:**
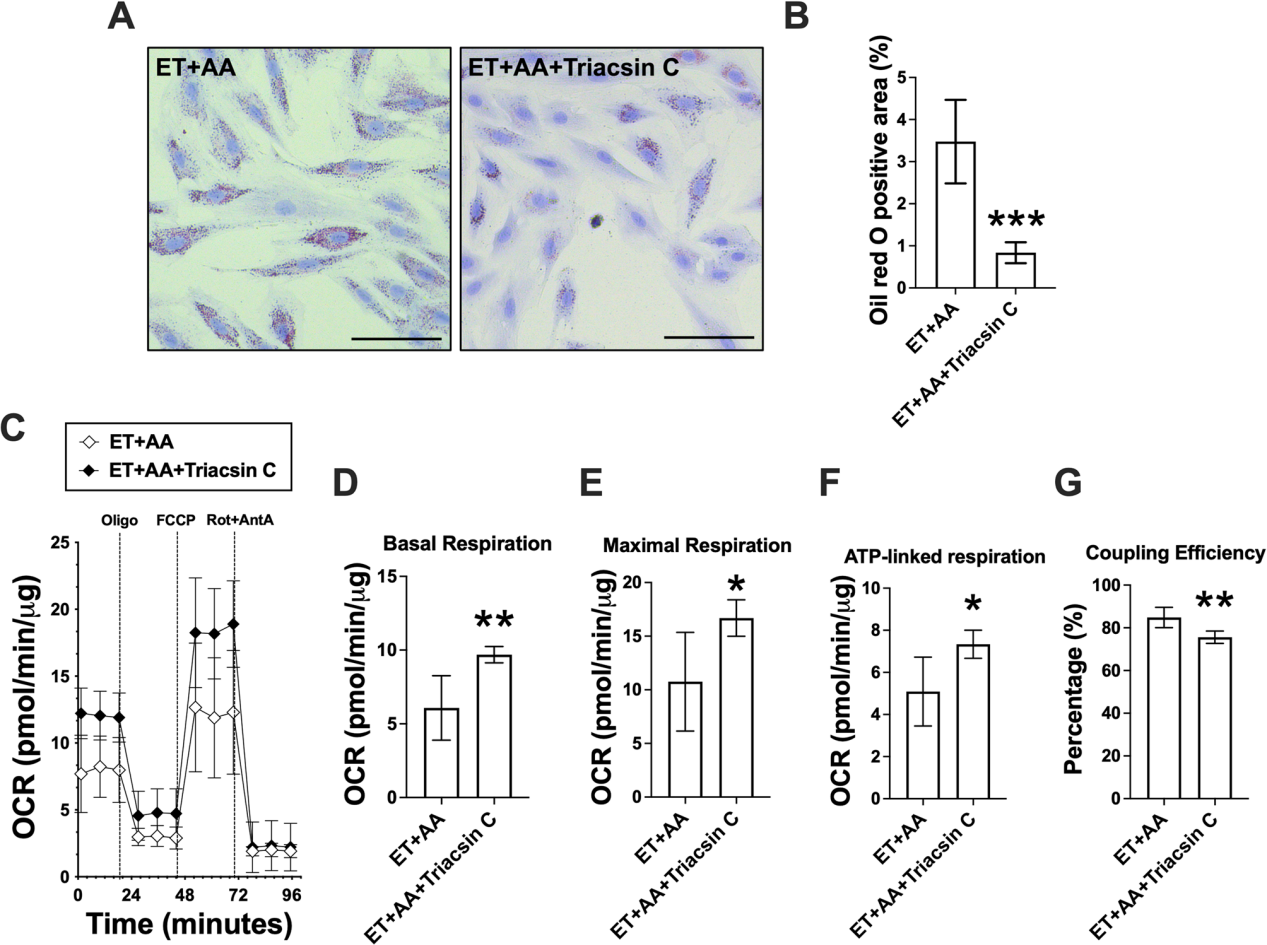
The effects of Triacsin C, an ACSL inhibitor, on lipid deposition, cell viability, and mitochondrial respiration in H9c2 cardiomyoblasts exposed to AA plus ethanol. **A** Representative pictures of oil-red O staining of H9c2 cells, scale bar = 100 μm. **B** Quantification of oil-red O staining, *n* = 6 per group. **C** Representative picture tracing OCR in H9c2 cells following sequential addition of oligomycin, FCCP, and rotenone/antimycin A. **D**-**G** Bar charts show the quantification of basal respiration, maximal respiration, ATP-linked respiration, and coupling efficiency, *n* = 5 wells per group. Values are expressed as mean ± SD, *P < 0.05, **P < 0.01

To determine the mechanism by which a combination of AA and ethanol promotes steatosis, H9c2 cardiomyoblasts were incubated in ET + AA with or without the treatment of Triacsin C, an inhibitor of long-chain acyl CoA synthases. In particular, ACSLs transform free FAs to fatty acyl CoAs which are then directed to synthetic or degradative pathways, including their esterification to triglycerides or phospholipids and their oxidation in mitochondria. In particular, Triacsin C is used to inhibit the production of AA-derived eicosanoids by preferential inhibiting arachidonoyl-CoA synthetase activity, leading to the inhibition of AA-mediated metabolic processes [34]. Triacsin C was reported to inhibit the formation of lipid droplets and improve mitochondrial metabolism in primary hepatocytes [35]. In the current study, Triacsin C treatment attenuated ET + AA-induced lipid accumulation in H9c2 cells as revealed by the oil-red O staining (*P* < 0.001, Fig. 8A and B). Next, to elucidate effects of Triacsin C on mitochondrial respiration, the Seahorse flux analysis was carried out (Fig. 8C) which showed that compared with ET + AA group, ET + AA + Triacsin C-treated cells showed an elevated cellular basal respiration (9.690 ± 0.5538 vs. 6.080 ± 2.182, *P* < 0.01, Fig. 8D), maximal respiration (16.69 ± 1.695 vs. 10.76 ± 4.599, *P* < 0.05, Fig. 8E), and ATP-linked respiration (7.336 ± 0.6673 vs. 5.090 ± 1.636, *P* < 0.05, Fig. 8F). On the other hand, ET + AA + Triacsin C decreased coupling efficiency in H9c2 cells compared with ET + AA-treated cells (*P* < 0.01, Fig. 8G). Although Triacsin C reduced steatosis and increased mitochondrial respiration, treatment with Triacsin C with or without ET and/or AA reduced the cell number (Suppl. Figure 1). However, markers of apoptosis were not altered by Triacsin C (not shown). These findings suggest that inhibiting ACSL activity by Triacsin C attenuated ET + AA-induced cell steatosis, increased basal and maximal respirations and ATP-linked respiration while decreasing coupling efficiency and cell proliferation.

## Discussion

In the present study, H9c2, a rat embryonic cardiomyoblast cell line, was used to analyze the cardiotoxicity of ethanol in combination with AA. AA potentiated ethanol-induced steatosis. Interestingly, AA by itself is beneficial for cardiomyoblast viability, and ET exposure abolished this positive effect. Moreover, AA supplementation by itself had no effect on oxidative stress but induced an elevation in H_2_O_2_ contents, a marker of oxidative stress, in combination with ethanol. RNA sequencing analysis revealed various pathways significantly altered upon ET + AA-treated cells, including ER stress. Further validation by real-time PCR and western blot analysis showed that ER stress markers are indeed increased in cells treated with ET + AA. In addition, ethanol in combination with AA reduced mitochondrial respiration. Further, to determine the relevance of these findings in vivo, a six-week chronic ethanol diet feeding model was used. It was demonstrated that ethanol exposure increased the AA level in myocardial phospholipids in rats. This increase in myocardial AA phospholipids correlated with the upregulation of key markers of ER stress and mitochondrial dysfunction, suggesting that altered AA metabolism played an important role in mediating early cardiac injury upon ethanol treatment. Further mechanistic studies in H9C2 cells revealed that inhibiting ACSLs by Triacsin C prevented ET + AA-induced steatosis and increased mitochondrial respiration and ATP-linked respiration. Overall, a combination of AA and ET leads to an increase in steatosis and ER stress and attenuates mitochondrial respiration. Our data also suggest that Triacsin C treatment inhibits steatosis but enhances mitochondrial respiration possibly via altered fatty acid partitioning between synthetic and oxidative processes.

It has been reported that AA has beneficial effects on myocyte growth [36]. Accordingly, AA alone at 50 µM concentration increased the number of H9c2 cells. Interestingly, this beneficial effect of AA is abolished by ethanol exposure. An interesting finding is that AA supplementation with ethanol increased cardiomyocyte steatosis. ACM is correlated with multiple detrimental histological and cellular changes in the myocardium. In particular, ethanol-induced intra-ventricular steatosis is mediated by diverse molecular mechanisms, including dysregulation of FA transport and lipid metabolism and upregulation of lipogenesis genes [14]. For example, chronic ethanol feeding in mice led to a decrease in ejection fraction and fraction shortening of the left ventricle, and it was associated with an increase in myocardial lipotoxicity. These mice exhibited up-regulated genes related to FA transport and *de novo* lipogenesis, such as cluster of differentiation 36 and stearoyl CoA desaturase 1 [15]. Studies have shown that dietary fatty acids exert significant effects on alcohol-induced steatosis. For example, Nanji AA et al. have reported that dietary linoleic acid, an omega-6 polyunsaturated fatty acid, is imperative to induce hepatic steatosis and inflammation in rats fed an ethanol diet [37]. Another in vivo study reported that AA aggravates ethanol-induced hepatic lipid accumulation [19]. The present study supports the direct effects of ET + AA in leading to steatosis in cardiomyoblasts. Ethanol treatment can increase FA uptake [15]. Thus, when cells are treated with ET + AA, the excess fatty acids taken up by cells, including AA, are redirected to storage as opposed to oxidation in mitochondria. Meanwhile, increased ethanol metabolism is known to inhibit mitochondrial fatty acid oxidation via depleting NAD + levels [14]. Ethanol-induced reduction in NAD + coupled with an impairment in mitochondrial respiration can also contribute, at least in part, to lipid accumulation in ET + AA-treated cells. In addition, AA has been shown to impair complex I and III during mitochondrial respiration [38]. Therefore, the direct effect of AA in inhibiting mitochondrial respiration, thereby increasing triglyceride accumulation, remains possible.

Of note, data from the present study provide interesting evidence that ET + AA-treated H9c2 cells exhibited an increase in ER stress markers. Ethanol has been demonstrated to induce ER stress, leading to multiple organ injuries [39]. Lu et al. have shown that ethanol-induced myocardial remodeling and suppressed ejection fraction are associated with ER stress by upregulating GRP78 and endoplasmic reticulum to nucleus signaling 1 (ERN1), a key sensor of unfolded proteins in the ER [40]. In addition, there is a tight association between AA and ER stress. Bae et al. have reported that incorporation of AA into membrane phospholipids gives rise to ER stress by increasing the levels of the spliced X-box binding protein 1 and phosphorylated eukaryotic translation initiation factor 2 subunit 1, thereby leading to apoptosis in human colon tumor cells [33]. In the present study, ethanol exposure elicited elevated AA-phospholipid levels with a concomitant increase in ER stress by upregulating GRP78 and ATF4 expression in the myocardium. These findings support significant roles of AA in promoting ethanol-induced myocardial injuries by inducing ER stress.

The protein level of ALDH2, a mitochondrial enzyme, is increased significantly in cells treated with ethanol alone or ET + AA. ALDH2 is the enzyme responsible for metabolizing acetaldehyde by consuming NAD+ [41]. ALDH1/2 enzymes are known to protect against oxidative stress. However, it should be pointed out that ethanol metabolism generates acetaldehyde and, therefore, the increase in ALDH2 can be attributed to a feedback regulation to promote acetaldehyde clearance. This notion can be supported by the observation that there is no alteration of 4-HNE, an aldehyde product of lipid peroxidation, in both ET and ET + AA-treated H9c2 cells, compared to the control. Moreover, H_2_O_2_ production was significantly increased in ET + AA-treated cells. Although ALDH1/2 enzymes help protect against oxidative stress, enhanced ALDH activity, particularly in the presence of increased acetaldehyde generation by ethanol treatment, can also promote oxidative stress. For example, ALDH2 knock-out mice exhibited a reduction in chronic ethanol-induced hepatic lipid peroxidation and inflammation [42, 43]. As mentioned, an increase in ALDH2 activity can deplete NAD+. Possibly, in the present study, an elevated NADH/NAD + ratio inhibits mitochondrial β-oxidation, thereby leading to steatosis and generation of reactive oxygen species in H9c2 cardiomyoblasts [44].

Another important finding is that ethanol led to increased AA-phospholipids content, which was associated with decreased NDUFB8 expression in rat myocardium. In addition, a combination of ethanol and AA reduced mitochondrial respiration in vitro. A decrease in mitochondrial enzyme activity is one of the adverse alterations found in myocardium with ACM [45]. For example, chronic ethanol-fed mice showed a decrease in ejection fraction and fraction shortening of the left ventricle, which was accompanied by elevated myocardial steatosis and down-regulated OXPHOS genes including *Ndufa8*, *Uqcrc2*, and *Sdhd* [15]. In a multi-omics study, cardiac spheroids exposed to chronic ethanol showed an increase in the production of AA and AA-derived metabolites, associated with a decrease in mitochondrial respiration and biosynthesis [16]. More interestingly, Cocco et al. using bovine heart mitochondria showed that AA inhibits complex I and III during mitochondrial respiration [38]. Le et al. have reported an elevated level of AA-containing phospholipids in heart failure [46]. This alteration is positively associated with mitochondrial dysfunction, lipid peroxide production, and overall cardiac impairment. In the present study, AA combined with ethanol led to compromised mitochondrial respiration through decreasing NDUFB8 protein level in H9c2 cells.

Mitochondria are one of the main sites producing H_2_O_2_ in response to the stress, and impairment of mitochondrial respiration leads to increased H_2_O_2_ production in H9c2 cells [47, 48]. Here, the present study provided evidence that H_2_O_2_ levels are increased in ET + AA-treated H9c2 cells, which is associated with ER stress and impaired mitochondrial respiration. H_2_O_2_ is strongly associated with mitochondrial dysfunction, oxidative stress, and ER stress in H9c2 cardiomyoblasts [49, 50]. For example, Yuvaraj et al. have reported that H_2_O_2_ leads to ER stress in H9c2 cardiomyoblasts through upregulating GRP78 and CHOP expressions [51]. Our data indicate that an increase in H_2_O_2_ production due to impaired mitochondrial respiration may play a role in promoting ER stress.

It is widely accepted that ethanol-induced steatosis is mediated via increased fatty acid uptake and lipogenesis. However, the free FAs need to be converted to fatty acyl CoAs before they are utilized as building blocks for triglycerides and phospholipids or for β-oxidation. ACSLs are enzymes involved in the synthesis of fatty acyl-CoAs. The present study showed that inhibiting ACSL activity, through Triascin C, significantly attenuated ET + AA treatment-induced steatosis, indicating that ET + AA-induced steatosis is mediated via ACSLs. However, Triacsin C increased mitochondrial respiration and ATP-linked respiration, presumably via Triacsin C-insensitive ACSL isoforms which redirects fatty acids to mitochondrial oxidation. Triacsin C can inhibit ACSL1, ACSL3, and ACSL4 [52]. Cardiomyocytes express other ACSLs, including 5 and 6 as well [53]. In particular, ACSL5 is shown to be located at the mitochondria, suggesting a role of this isoform in facilitating entry of fatty acids into β-oxidation [54]. Thus, it is possible that AA delivery to mitochondria may be increased by ACSL5 which is not inhibited by Triacsin C, which, in turn, can increase mitochondrial respiration in ET + AA + Triacsin C-treated cells.

The current study also showed that inhibition of ACSLs by Triacsin C led to decreased cell numbers regardless of treatment conditions. These effects of Triacsin C on cell viability were attributed to the inhibition of cell proliferation due to a reduction in partitioning of cellular FAs to the phospholipid pool. This concept is substantiated by the evidence that phospholipids are essential for the viability of cardiomyocytes [55, 56]. In fact, previous studies have shown that Triacsin C inhibited the synthesis of phospholipids and cell proliferation [57, 58]. As mentioned, Triacsin C inhibits both ACSL1 and 3 which can direct fatty acids to phospholipids [52].

Our findings are clinically relevant. For example, polyunsaturated fatty acids are required to promote alcohol-induced organ injuries [37, 59]. Of note, linoleic acid (LA), an ω−6 polyunsaturated fatty acid, is a precursor for AA. LA is required for inducing ethanol-induced liver injury by increasing the formation of oxidized LA metabolites [60]. LA can be converted to AA by desaturases and elongases. Specifically, increased delta-6-desaturase activity is found in hypertension patients, promoting the conversion of LA to AA and subsequent pro-inflammatory effects [46]. Pharmacological inhibition of delta-6-desaturase ameliorates impaired mitochondrial function, oxidative stress, and inflammation in heart failure animals by decreasing myocardial content of AA-phospholipids [46]. Moreover, the cellular levels of AA and AA-derived metabolites are increased in cardiomyocytes in response to ethanol exposure [16]. In particular, a clinical study in human subjects with alcohol-related hypertension has reported a positive correlation between alcohol consumption and excretion of urine 20-hydroxyeicosatetraenoic acid (HETE), an AA-derived metabolite [61]. Emerging studies have reported that 20-HETE is a vasoconstrictor that mediates hypertension by increasing renal and peripheral vascular resistance [62]. Han et al. have found that 20-HETE exerts adverse effects on H9c2 cardiomyoblasts by increasing reactive oxygen species production and mitochondrial damage [63]. Given the potential role of HETE signaling in CVD, future investigations are warranted to elucidate the impact of AA supplementation in promoting the formation of HETE and other lipid mediators by pharmacological targeting of enzymes such as lipoxygenases and cytochromes P450. Additionally, other fatty acids, including oleic acid and dihomo-γ-linolenic acid, exert an important role for cardiovascular health [64, 65]. Therefore, the role of other FAs in mediating ACM cannot be ruled out. Although AA has some favorable effects in improving cardiomyocyte functions [6], the presence of ethanol can negate such benefits. Evidence suggests that ethanol in combination with a high fat diet aggravates organ injury [66]. Our findings suggest that a diet rich in AA or linoleic acid, which can increase AA levels in the body, can enhance ethanol-induced cardiomyocyte injury.

The present study has some limitations. For example, an in vivo ACM model using an ethanol diet supplemented with AA is warranted to assess the role of dietary AA supplementation in enhancing alcohol-induced abnormal cardiac performance and hemodynamics, and structural changes of the heart. In addition, ATP-linked respiration and coupling efficiency, as assessed by the Seahorse assay, provide an estimate of ATP synthesis inferred from oxygen consumption rates in response to oligomycin treatment, rather than a direct measurement of ATP level in the cells. Moreover, measurement of the precise ET concentration in the myocardium is challenging. Currently, no in vitro model fully recapitulates effects of alcohol in the heart. However, organ-organ interaction might exert a pivotal role in ACM. The liver mainly metabolizes alcohol, leading to the production of acetaldehyde, a key factor inducing cardiac dysfunction [67]. While H9c2 cells provide a robust model to study molecular pathways, they may not fully recapitulate all aspects of mature cardiomyocyte biology [68]. Isolation of primary cardiomyocytes from adult rats or mice exposed to an ethanol diet supplemented with AA can be a more reliable model to uncover the underlying molecular mechanisms of ACM.

## Conclusions

The present study indicates that chronic alcohol exposure elicits elevated AA-phospholipid levels, which was associated with molecular and cellular changes, including an increase in ER stress and a decrease in mitochondrial respiration. AA supplementation potentiates ethanol-induced steatosis, ER stress, and mitochondrial dysfunction in H9c2 cardiomyoblasts. The present findings also suggest that ET + AA-induced steatosis is mediated by ACSLs. Finally, the data indicate that inhibition of Triacsin C-sensitive ACSLs prevents steatosis but increases mitochondrial respiration due to altered fatty acid partitioning to synthetic versus oxidation reactions. Taken together, this study suggests that avoiding a diet rich in ω−6 polyunsaturated FAs during ethanol intake may mitigate potential cardiovascular risks.

## Supplementary Information

Supplementary Material 1.

Supplementary Material 2.

## Data Availability

Data available on request from the authors.

## Abbreviations

4-HNE: 4-Hydroxynonenal
AA: Arachidonic acid
ACM: Alcohol-associated cardiomyopathy
ACSL: Long-chain fatty acyl-CoA synthetase
ALDH1/2: Aldehyde dehydrogenases 1/2
ATF4: Activating transcription factor 4
CHOP: C/EBP homologous protein
COX I: Cytochrome C oxidase I
CVD: Cardiovascular disease
DMEM: Dulbecco’s Modified Eagle’s Medium
ER: Endoplasmic reticulum
FA: Fatty acid
FBS: Fetal bovine serum
FCCP: Carbonyl cyanide 4-(trifluoromethoxy) phenylhydrazone
GC: Gas chromatography
GRP78: Glucose-regulated protein 78
HETE: hydroxyeicosatetraenoic acid
IPA: Ingenuity pathway analysis
LA: linoleic acid
NDHFB8: NADH: ubiquinone oxidoreductase subunit B8
OCR: Oxygen consumption rate
OXPHOS: Oxidative phosphorylation
SD: Standard deviation
SDHB: Succinate dehydrogenase complex iron sulfur subunit B
TG: Triglyceride
UQCRC2: Ubiquinol-cytochrome C reductase core protein 2
