# Supplementary material for: A combination of ethanol and arachidonic acid promotes steatosis and endoplasmic reticulum stress and impairs mitochondrial respiration in H9c2 cardiomyoblasts
Source: Lipids Health Dis. 2025 Dec 16;24:385. doi: 10.1186/s12944-025-02792-3 (PMC12709855; doi:10.1186/s12944-025-02792-3)
Supplement: Supplementary file 1 — Supplementary Material 1. [file 12944_2025_2792_MOESM1_ESM.docx]

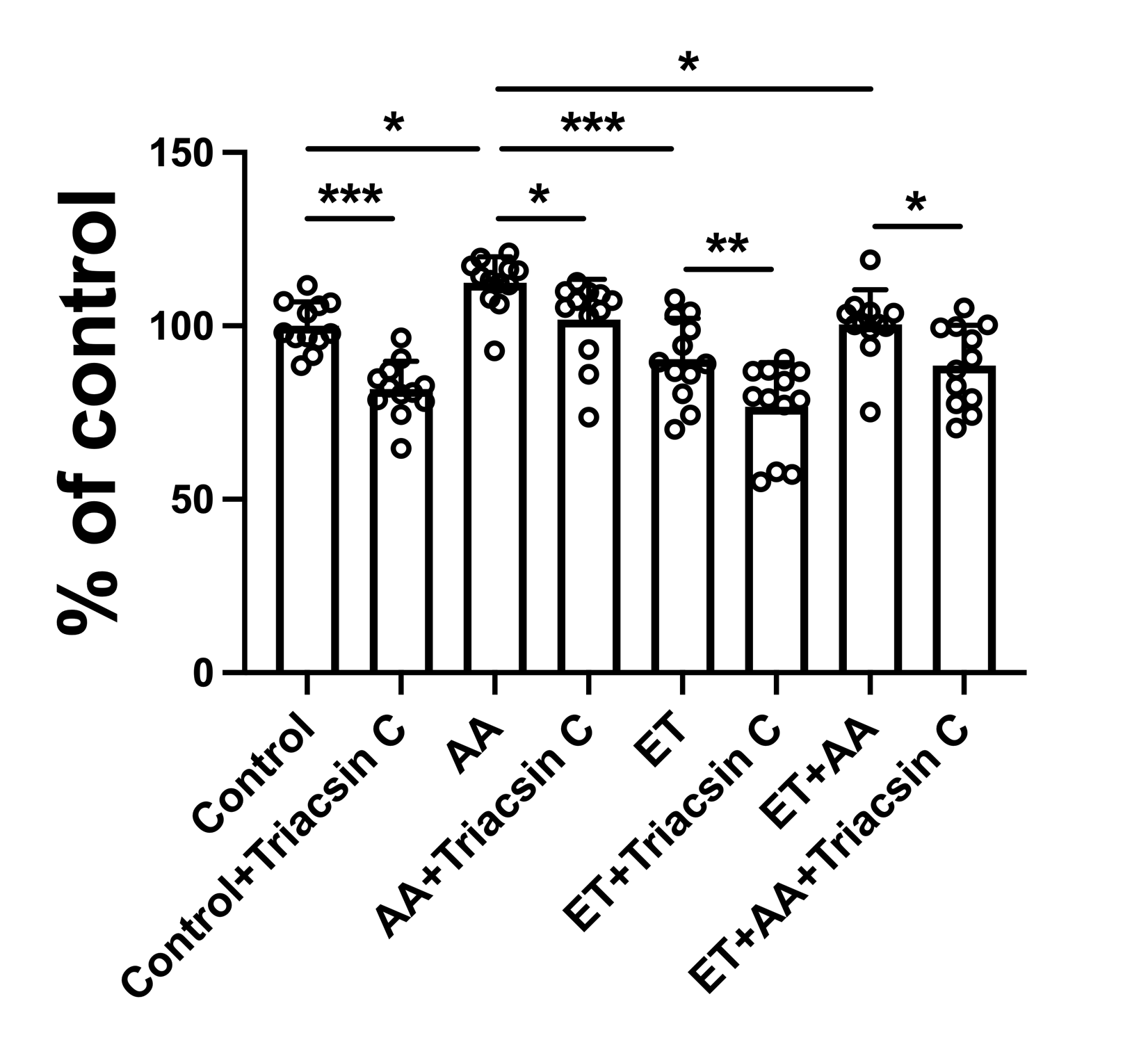


**Suppl. Figure 1. The effects of Triacsin C on cell viability of H9c2 cells.** Cell viability was evaluated by PrestoBlue assay. n = 12 per group. Values are expressed as mean ± SD, **P* < 0.05, ***P* < 0.01, ****P* < 0.001.
