# Supplementary material for: A combination of ethanol and arachidonic acid promotes steatosis and endoplasmic reticulum stress and impairs mitochondrial respiration in H9c2 cardiomyoblasts
Source: Lipids Health Dis. 2025 Dec 16;24:385. doi: 10.1186/s12944-025-02792-3 (PMC12709855; doi:10.1186/s12944-025-02792-3)
Supplement: Supplementary file 2 — Supplementary Material 2. [file 12944_2025_2792_MOESM2_ESM.docx]

**Suppl. Table 1**

| Gene name | log2(Fold Change) | *P* value | Adjusted *P* value |
| --- | --- | --- | --- |
| Cpt1a | 1.748839 | 5.47E-77 | 7.62E-73 |
| Angptl4 | 3.904179 | 2.26E-46 | 1.57E-42 |
| Abca1 | -2.52115 | 3.22E-32 | 1.50E-28 |
| Tnnt2 | -1.17038 | 2.19E-31 | 7.62E-28 |
| Mcm4 | -1.07009 | 1.67E-24 | 4.66E-21 |
| Il1rl1 | -1.2715 | 2.55E-24 | 5.90E-21 |
| Cyp51 | 0.781004 | 6.32E-22 | 1.26E-18 |
| Uhrf1 | -0.92513 | 6.26E-21 | 1.09E-17 |
| Pole | -1.06799 | 4.47E-20 | 6.91E-17 |
| Dhcr24 | 1.110863 | 1.24E-19 | 1.73E-16 |
| Ptgr1 | 1.142666 | 1.07E-18 | 1.36E-15 |
| Msmo1 | 0.834764 | 5.01E-18 | 5.82E-15 |
| Lmnb1 | -1.04862 | 6.79E-18 | 7.27E-15 |
| Trib3 | 1.312223 | 9.07E-18 | 9.02E-15 |
| Mcm5 | -0.96846 | 7.91E-17 | 7.34E-14 |
| Nqo1 | 1.185673 | 1.05E-16 | 9.17E-14 |
| Mcm3 | -0.94899 | 3.14E-16 | 2.57E-13 |
| Gadd45a | 0.74537 | 1.64E-15 | 1.27E-12 |
| Clspn | -1.32235 | 2.92E-15 | 1.98E-12 |
| LOC100909521 | 0.771011 | 3.03E-15 | 1.98E-12 |
| Cdk2 | -0.88484 | 3.13E-15 | 1.98E-12 |
| Slc6a9 | 0.836595 | 2.94E-15 | 1.98E-12 |
| Blm | -1.03075 | 1.46E-14 | 8.82E-12 |
| Mcm10 | -1.05278 | 1.70E-14 | 9.86E-12 |
| Casp4 | 1.006391 | 2.32E-14 | 1.29E-11 |
| Topbp1 | -0.85601 | 4.22E-14 | 2.26E-11 |
| Mdc1 | -0.82952 | 4.72E-14 | 2.43E-11 |
| Hells | -1.26298 | 4.90E-14 | 2.44E-11 |
| Dtl | -1.0539 | 7.12E-14 | 3.31E-11 |
| LOC100910708 | 1.565561 | 6.99E-14 | 3.31E-11 |
| Atad2 | -0.96786 | 9.42E-14 | 4.23E-11 |
| Fads2 | 0.777225 | 1.03E-13 | 4.47E-11 |
| Idh1 | 0.717727 | 1.49E-13 | 6.20E-11 |
| Dnmt1 | -0.84887 | 1.52E-13 | 6.20E-11 |
| Plin2 | 0.715465 | 3.42E-13 | 1.36E-10 |
| Pola1 | -0.78733 | 4.97E-13 | 1.92E-10 |
| Slc38a4 | 0.801074 | 5.25E-13 | 1.98E-10 |
| Ccne2 | -0.90655 | 5.59E-13 | 2.05E-10 |
| Kif11 | -0.77703 | 5.91E-13 | 2.11E-10 |
| Cep55 | -0.77234 | 7.51E-13 | 2.61E-10 |
| Dnm1 | 1.111938 | 1.27E-12 | 4.30E-10 |
| Anln | -0.79755 | 1.34E-12 | 4.44E-10 |
| Ctsl | 0.658274 | 1.40E-12 | 4.53E-10 |
| Gsta1 | 1.821963 | 1.57E-12 | 4.97E-10 |
| Acaa2 | 1.185113 | 1.68E-12 | 5.18E-10 |
| Ivns1abp | -0.75755 | 1.91E-12 | 5.78E-10 |
| Akr1b8 | 1.333971 | 1.98E-12 | 5.87E-10 |
| Hmox1 | 0.72404 | 2.02E-12 | 5.87E-10 |
| Xirp1 | -0.81148 | 2.49E-12 | 7.07E-10 |
| Nrn1 | 0.649755 | 2.64E-12 | 7.34E-10 |
| Arrdc3 | 0.840701 | 4.63E-12 | 1.26E-09 |
| Fads1 | 0.61201 | 7.79E-12 | 2.09E-09 |
| Hnrnpa1 | -0.52796 | 8.58E-12 | 2.25E-09 |
| Tnnt1 | -0.90037 | 9.30E-12 | 2.40E-09 |
| Gtse1 | -0.61435 | 1.02E-11 | 2.59E-09 |
| Ccnj | -8.37106 | 1.04E-11 | 2.60E-09 |
| Timeless | -0.95786 | 1.13E-11 | 2.75E-09 |
| Errfi1 | -0.70077 | 1.31E-11 | 3.14E-09 |
| Dusp27 | -0.68872 | 2.12E-11 | 4.94E-09 |
| Bard1 | -0.88325 | 2.13E-11 | 4.94E-09 |
| Espl1 | -0.75245 | 2.36E-11 | 5.38E-09 |
| Mycn | -0.86544 | 2.51E-11 | 5.63E-09 |
| E2f7 | -1.07888 | 2.69E-11 | 5.95E-09 |
| Prc1 | -0.55644 | 3.56E-11 | 7.75E-09 |
| Dek | -0.95079 | 3.64E-11 | 7.80E-09 |
| Chaf1a | -0.80591 | 4.15E-11 | 8.75E-09 |
| Dhcr7 | 0.954689 | 7.01E-11 | 1.46E-08 |
| Wdhd1 | -1.00629 | 9.02E-11 | 1.85E-08 |
| Myh3 | -1.10691 | 1.24E-10 | 2.51E-08 |
| Tmpo | -0.88335 | 2.02E-10 | 4.01E-08 |
| Ifi27l2b | 1.403831 | 2.32E-10 | 4.49E-08 |
| Synpo2l | -0.66096 | 2.29E-10 | 4.49E-08 |
| Wee1 | -0.89305 | 2.50E-10 | 4.76E-08 |
| Ticrr | -1.1055 | 2.63E-10 | 4.82E-08 |
| Ptprn | 0.683955 | 2.63E-10 | 4.82E-08 |
| Lmnb2 | -0.63948 | 2.61E-10 | 4.82E-08 |
| Kcnj8 | 0.812311 | 2.75E-10 | 4.98E-08 |
| E2f8 | -1.24076 | 3.06E-10 | 5.47E-08 |
| Spdl1 | -0.90288 | 3.76E-10 | 6.63E-08 |
| Spp1 | 0.651663 | 3.89E-10 | 6.78E-08 |
| Kif20b | -0.85594 | 4.93E-10 | 8.47E-08 |
| Incenp | -0.74789 | 5.62E-10 | 9.54E-08 |
| Kif23 | -0.61558 | 5.79E-10 | 9.70E-08 |
| Btg1 | 0.525405 | 6.11E-10 | 1.01E-07 |
| Mcm6 | -0.8347 | 6.21E-10 | 1.02E-07 |
| Sgol2 | -0.67765 | 7.11E-10 | 1.15E-07 |
| Gas7 | 0.857948 | 7.95E-10 | 1.27E-07 |
| Ptprq | -1.49821 | 8.43E-10 | 1.33E-07 |
| Gng8 | 1.103013 | 8.97E-10 | 1.40E-07 |
| Rbl1 | -0.81006 | 9.15E-10 | 1.41E-07 |
| Sqle | 0.532735 | 1.16E-09 | 1.78E-07 |
| Slc29a1 | 0.648825 | 1.23E-09 | 1.87E-07 |
| AC114502.1 | -7.7172 | 1.26E-09 | 1.89E-07 |
| Brca1 | -0.94791 | 1.43E-09 | 2.12E-07 |
| Ckap2l | -0.60627 | 1.50E-09 | 2.18E-07 |
| Hirip3 | -0.96335 | 1.50E-09 | 2.18E-07 |
| Aig1 | 0.861713 | 1.62E-09 | 2.32E-07 |
| Zfp367 | -1.10039 | 2.02E-09 | 2.87E-07 |
| Cdt1 | -0.78675 | 2.19E-09 | 3.08E-07 |
| Rmnd5b | 7.435995 | 2.68E-09 | 3.72E-07 |
| Bub1b | -0.5668 | 2.73E-09 | 3.72E-07 |
| Exo1 | -0.89162 | 2.70E-09 | 3.72E-07 |
| Smc2 | -0.88221 | 3.30E-09 | 4.46E-07 |
| Fdps | 1.120311 | 3.43E-09 | 4.59E-07 |
| Ncapd3 | -0.80212 | 3.52E-09 | 4.63E-07 |
| Dscc1 | -0.82454 | 3.50E-09 | 4.63E-07 |
| Nasp | -0.69134 | 3.67E-09 | 4.77E-07 |
| Ftl1 | 0.995587 | 4.05E-09 | 5.22E-07 |
| Mcm2 | -0.66551 | 4.31E-09 | 5.50E-07 |
| Fancd2 | -0.60588 | 4.85E-09 | 6.14E-07 |
| Acot2 | 0.862405 | 4.96E-09 | 6.17E-07 |
| Fdft1 | 0.719132 | 4.95E-09 | 6.17E-07 |
| Spag5 | -0.59084 | 5.25E-09 | 6.42E-07 |
| Il4r | 0.61369 | 5.26E-09 | 6.42E-07 |
| Esco2 | -0.8399 | 5.69E-09 | 6.88E-07 |
| Syt13 | -0.66121 | 6.28E-09 | 7.54E-07 |
| Arhgap11a | -0.83885 | 6.38E-09 | 7.59E-07 |
| Nup107 | -0.54642 | 6.50E-09 | 7.67E-07 |
| Iqgap3 | -0.56291 | 6.89E-09 | 8.06E-07 |
| Oxct1 | -0.59078 | 7.32E-09 | 8.49E-07 |
| Lnx1 | 1.048643 | 7.80E-09 | 8.97E-07 |
| Fen1 | -0.70479 | 1.03E-08 | 1.17E-06 |
| Stil | -0.78555 | 1.04E-08 | 1.17E-06 |
| Tonsl | -1.07185 | 1.14E-08 | 1.28E-06 |
| Mcm8 | -0.97733 | 1.15E-08 | 1.28E-06 |
| Cdc6 | -0.97539 | 1.36E-08 | 1.50E-06 |
| Dysf | 0.478516 | 1.43E-08 | 1.57E-06 |
| Tm7sf2 | 1.032516 | 1.57E-08 | 1.71E-06 |
| Nkd2 | -0.64221 | 1.67E-08 | 1.80E-06 |
| Kif18b | -0.73618 | 1.86E-08 | 1.99E-06 |
| Unc93b1 | 0.732112 | 1.92E-08 | 2.04E-06 |
| Rrm1 | -0.58333 | 1.95E-08 | 2.06E-06 |
| Brip1 | -1.0283 | 2.23E-08 | 2.33E-06 |
| Slc16a14 | 1.380961 | 2.31E-08 | 2.38E-06 |
| Dlgap5 | -0.55124 | 2.32E-08 | 2.38E-06 |
| Ucp2 | 0.943832 | 2.34E-08 | 2.38E-06 |
| Cenpi | -0.64928 | 2.34E-08 | 2.38E-06 |
| Polq | -1.08462 | 2.44E-08 | 2.46E-06 |
| Krt7 | -1.01848 | 2.71E-08 | 2.71E-06 |
| Gstp1 | 1.228418 | 2.81E-08 | 2.78E-06 |
| Prnp | 0.444638 | 2.82E-08 | 2.78E-06 |
| Atad5 | -1.22634 | 2.89E-08 | 2.84E-06 |
| Tacc3 | -0.53546 | 3.08E-08 | 3.00E-06 |
| Aif1l | -0.61621 | 3.52E-08 | 3.41E-06 |
| Igh-1a | -0.92054 | 3.69E-08 | 3.54E-06 |
| Cd151 | 1.409714 | 3.97E-08 | 3.72E-06 |
| Ncaph | -0.63812 | 3.93E-08 | 3.72E-06 |
| Atf5 | 0.702025 | 3.98E-08 | 3.72E-06 |
| Fbxo5 | -0.83463 | 3.94E-08 | 3.72E-06 |
| Insig1 | 0.53454 | 4.27E-08 | 3.96E-06 |
| Chac1 | 0.639069 | 4.81E-08 | 4.44E-06 |
| Tcof1 | -0.60807 | 5.02E-08 | 4.60E-06 |
| Tmem63a | 0.621444 | 5.14E-08 | 4.68E-06 |
| Msh2 | -0.63433 | 5.18E-08 | 4.68E-06 |
| Fanca | -0.72526 | 5.23E-08 | 4.70E-06 |
| Xdh | 0.511868 | 5.43E-08 | 4.84E-06 |
| E2f2 | -0.79886 | 5.88E-08 | 5.21E-06 |
| Foxm1 | -0.69088 | 6.38E-08 | 5.62E-06 |
| AABR07049961.1 | -1.19198 | 7.16E-08 | 6.23E-06 |
| Ezh2 | -0.59422 | 7.21E-08 | 6.23E-06 |
| Nsdhl | 0.645771 | 7.17E-08 | 6.23E-06 |
| Kpnb1 | -0.4363 | 7.28E-08 | 6.26E-06 |
| Tkt | 0.808983 | 8.17E-08 | 6.98E-06 |
| Ccna2 | -0.61431 | 8.40E-08 | 7.13E-06 |
| Fignl1 | -1.05688 | 9.25E-08 | 7.80E-06 |
| Troap | -0.65195 | 1.06E-07 | 8.91E-06 |
| Ddit3 | 0.651637 | 1.09E-07 | 9.07E-06 |
| Top2a | -0.79968 | 1.09E-07 | 9.07E-06 |
| Scn1b | 1.019946 | 1.25E-07 | 1.02E-05 |
| Slc25a1 | 0.858737 | 1.24E-07 | 1.02E-05 |
| Mcm7 | -0.80766 | 1.34E-07 | 1.09E-05 |
| Fanci | -0.64403 | 1.37E-07 | 1.11E-05 |
| Gsr | 0.589535 | 1.39E-07 | 1.12E-05 |
| Ncapg2 | -0.85062 | 1.41E-07 | 1.13E-05 |
| Lig1 | -0.57115 | 1.54E-07 | 1.22E-05 |
| Kif24 | -1.21013 | 1.68E-07 | 1.33E-05 |
| Snx10 | 0.679673 | 1.71E-07 | 1.34E-05 |
| Hmgb2 | -0.59162 | 1.83E-07 | 1.43E-05 |
| Mroh1 | 0.542475 | 2.09E-07 | 1.63E-05 |
| Ggt1 | 1.47306 | 2.16E-07 | 1.67E-05 |
| Sqstm1 | 0.615446 | 2.24E-07 | 1.72E-05 |
| Kif14 | -0.86444 | 2.45E-07 | 1.86E-05 |
| LOC679582 | -0.77012 | 2.44E-07 | 1.86E-05 |
| Gatm | -0.47858 | 2.50E-07 | 1.89E-05 |
| Mki67 | -1.04825 | 2.56E-07 | 1.93E-05 |
| Msh6 | -0.58468 | 2.60E-07 | 1.94E-05 |
| Decr1 | 0.809812 | 3.11E-07 | 2.31E-05 |
| Anlnl1 | -0.75573 | 3.19E-07 | 2.36E-05 |
| Sfxn1 | 0.628628 | 3.23E-07 | 2.38E-05 |
| Ctsf | 0.592735 | 3.48E-07 | 2.55E-05 |
| Slc52a2 | 0.738067 | 4.05E-07 | 2.95E-05 |
| LOC100360087 | 0.918843 | 4.64E-07 | 3.36E-05 |
| Fosl1 | 0.545885 | 4.86E-07 | 3.50E-05 |
| Mt1 | 2.680479 | 4.89E-07 | 3.51E-05 |
| Aqp1 | -0.77944 | 4.92E-07 | 3.51E-05 |
| Smc4 | -0.59658 | 5.16E-07 | 3.67E-05 |
| G2e3 | -0.71037 | 5.39E-07 | 3.81E-05 |
| Stbd1 | 0.485308 | 5.53E-07 | 3.85E-05 |
| Herpud1 | 0.601412 | 5.51E-07 | 3.85E-05 |
| Kntc1 | -0.73564 | 5.50E-07 | 3.85E-05 |
| Casc5 | -0.82632 | 5.73E-07 | 3.97E-05 |
| Recql4 | -0.65646 | 5.82E-07 | 3.99E-05 |
| Kif4a | -0.593 | 5.78E-07 | 3.99E-05 |
| Prr11 | -0.9663 | 5.88E-07 | 4.01E-05 |
| Orc1 | -0.94973 | 5.98E-07 | 4.04E-05 |
| Tgfb1 | 0.587294 | 5.95E-07 | 4.04E-05 |
| Car3 | 0.462507 | 6.12E-07 | 4.11E-05 |
| Donson | -0.83766 | 6.31E-07 | 4.22E-05 |
| G6pdx | 0.640081 | 6.40E-07 | 4.26E-05 |
| Ech1 | 0.898282 | 6.81E-07 | 4.52E-05 |
| Odc1 | 0.682373 | 7.30E-07 | 4.82E-05 |
| Gas2l3 | -0.75586 | 7.38E-07 | 4.84E-05 |
| Cdc7 | -0.86036 | 7.45E-07 | 4.85E-05 |
| Srpx2 | 0.732872 | 7.50E-07 | 4.85E-05 |
| Mfsd3 | 0.769292 | 7.47E-07 | 4.85E-05 |
| AABR07000121.1 | -0.72985 | 7.64E-07 | 4.93E-05 |
| Fam111a | -0.61415 | 8.10E-07 | 5.17E-05 |
| Arhgap19 | -0.62729 | 8.06E-07 | 5.17E-05 |
| Prdx5 | 0.863622 | 8.23E-07 | 5.23E-05 |
| Pou2f2 | -1.30032 | 9.21E-07 | 5.82E-05 |
| Ninj1 | 0.778698 | 9.38E-07 | 5.91E-05 |
| Lmo7 | -0.69648 | 9.45E-07 | 5.93E-05 |
| Acadl | 0.61406 | 9.62E-07 | 6.00E-05 |
| Brca2 | -1.20362 | 1.01E-06 | 6.29E-05 |
| Sat1 | 0.657249 | 1.02E-06 | 6.29E-05 |
| Eri1 | -0.52881 | 1.05E-06 | 6.46E-05 |
| Olfm2 | 0.421654 | 1.09E-06 | 6.70E-05 |
| Cenpu | -0.67291 | 1.14E-06 | 6.94E-05 |
| Cdca2 | -0.55671 | 1.15E-06 | 6.98E-05 |
| Ncapd2 | -0.46158 | 1.18E-06 | 7.15E-05 |
| Depdc1 | -0.68668 | 1.34E-06 | 8.05E-05 |
| Mxd4 | 0.527128 | 1.34E-06 | 8.05E-05 |
| Kif15 | -0.71405 | 1.41E-06 | 8.43E-05 |
| Hmgn5b | -1.14204 | 1.42E-06 | 8.46E-05 |
| Lrrcc1 | -0.61953 | 1.48E-06 | 8.75E-05 |
| Slc48a1 | 0.653283 | 1.49E-06 | 8.76E-05 |
| Ints7 | -0.45308 | 1.49E-06 | 8.77E-05 |
| Anp32e | -0.62633 | 1.52E-06 | 8.91E-05 |
| Parpbp | -0.62622 | 1.70E-06 | 9.92E-05 |
| Srsf2 | -0.50876 | 1.71E-06 | 9.93E-05 |
| Tgfbi | 0.57413 | 1.73E-06 | 9.96E-05 |
| Pask | -1.01538 | 1.78E-06 | 0.000102 |
| LOC100911874 | 0.936712 | 1.77E-06 | 0.000102 |
| Smim3 | 0.510921 | 1.78E-06 | 0.000102 |
| Hat1 | -0.51185 | 1.95E-06 | 0.00011 |
| Slc3a2 | 0.454742 | 1.94E-06 | 0.00011 |
| Pola2 | -0.56623 | 2.08E-06 | 0.000117 |
| Dgat2 | 0.672332 | 2.18E-06 | 0.000122 |
| RGD1306058 | 0.711956 | 2.19E-06 | 0.000122 |
| Serpinf1 | 0.47629 | 2.23E-06 | 0.000124 |
| Gen1 | -0.86125 | 2.23E-06 | 0.000124 |
| Cdkn2b | 0.506623 | 2.35E-06 | 0.00013 |
| Ccnf | -0.45808 | 2.55E-06 | 0.00014 |
| Racgap1 | -0.50258 | 2.54E-06 | 0.00014 |
| Nup160 | -0.68334 | 2.68E-06 | 0.000146 |
| Haus6 | -0.77791 | 2.78E-06 | 0.000151 |
| Kif1b | 0.486844 | 2.77E-06 | 0.000151 |
| Fam20c | 0.365602 | 2.84E-06 | 0.000152 |
| Fads3 | 0.548677 | 2.84E-06 | 0.000152 |
| Fth1 | 0.901498 | 2.82E-06 | 0.000152 |
| Taldo1 | 0.716209 | 2.94E-06 | 0.000157 |
| Sgk1 | 0.634619 | 2.97E-06 | 0.000157 |
| Eme1 | -0.96286 | 2.97E-06 | 0.000157 |
| Larp7 | -0.50524 | 3.02E-06 | 0.000159 |
| Fgf7 | 0.637587 | 3.08E-06 | 0.000162 |
| Rad51 | -0.62348 | 3.10E-06 | 0.000162 |
| Gins3 | -0.72293 | 3.29E-06 | 0.000171 |
| Hjurp | -0.52959 | 3.28E-06 | 0.000171 |
| N4bp2l1 | 1.263921 | 3.49E-06 | 0.00018 |
| Afap1l2 | -0.66432 | 3.48E-06 | 0.00018 |
| Aspm | -1.01797 | 3.57E-06 | 0.000184 |
| Cyp2d4 | 1.265052 | 3.62E-06 | 0.000185 |
| Pink1 | 0.63306 | 3.66E-06 | 0.000187 |
| Fam83d | -0.51479 | 3.71E-06 | 0.000188 |
| Tap1 | 0.746495 | 3.82E-06 | 0.000193 |
| Akap12 | -0.65974 | 3.83E-06 | 0.000193 |
| Ercc6l | -0.62066 | 3.87E-06 | 0.000194 |
| Atp8a2 | 1.103774 | 4.03E-06 | 0.000202 |
| Arpc1b | 0.503175 | 4.06E-06 | 0.000202 |
| Dsn1 | -0.49235 | 4.05E-06 | 0.000202 |
| Rad21 | -0.4052 | 4.09E-06 | 0.000203 |
| Fubp1 | -0.42849 | 4.14E-06 | 0.000204 |
| Phf19 | -0.71153 | 4.23E-06 | 0.000208 |
| Scd2 | -0.55763 | 4.35E-06 | 0.000213 |
| Fancm | -0.85626 | 4.56E-06 | 0.000223 |
| RGD1310335 | -0.73016 | 4.78E-06 | 0.000233 |
| Ect2 | -0.51661 | 4.88E-06 | 0.000237 |
| Il17d | 1.909579 | 5.03E-06 | 0.000243 |
| Mms22l | -0.56954 | 5.08E-06 | 0.000244 |
| Cep72 | -0.68871 | 5.17E-06 | 0.000248 |
| Vopp1 | 0.520199 | 5.23E-06 | 0.00025 |
| Lrp10 | 0.408858 | 5.34E-06 | 0.000254 |
| Psph | 0.796566 | 5.49E-06 | 0.000261 |
| Tsku | 0.452116 | 5.55E-06 | 0.000263 |
| Atf3 | 1.093328 | 5.59E-06 | 0.000264 |
| Dbp | 0.675883 | 6.56E-06 | 0.000308 |
| Cenph | -0.75458 | 6.95E-06 | 0.000325 |
| Tsc22d3 | 0.623747 | 6.95E-06 | 0.000325 |
| Kcnk12 | 0.663574 | 7.02E-06 | 0.000326 |
| Tspan17 | 0.845433 | 7.01E-06 | 0.000326 |
| Srsf1 | -0.48806 | 7.74E-06 | 0.000358 |
| Ddx11 | -0.73727 | 7.98E-06 | 0.000368 |
| Sesn2 | 0.452894 | 9.08E-06 | 0.000417 |
| Rad54b | -1.13601 | 9.16E-06 | 0.00042 |
| LOC690422 | -0.52153 | 9.27E-06 | 0.000423 |
| Fam3a | 0.493221 | 9.40E-06 | 0.000428 |
| Tinagl1 | -0.4996 | 9.65E-06 | 0.000437 |
| Tsc22d1 | 0.521156 | 1.01E-05 | 0.000455 |
| Ddias | -0.63661 | 1.10E-05 | 0.000495 |
| Tgfb3 | -0.33133 | 1.14E-05 | 0.00051 |
| Gmnn | -0.63282 | 1.15E-05 | 0.000515 |
| LOC100911693 | -0.50979 | 1.16E-05 | 0.000518 |
| LOC100910526 | 0.686108 | 1.17E-05 | 0.000522 |
| Bub1 | -0.44506 | 1.20E-05 | 0.000531 |
| Nnmt | 0.751119 | 1.34E-05 | 0.000591 |
| Klhl8 | 0.765386 | 1.36E-05 | 0.000597 |
| Tead4 | -0.54869 | 1.36E-05 | 0.000597 |
| Tmem19 | 0.730159 | 1.37E-05 | 0.000598 |
| Dnajc9 | -0.48211 | 1.41E-05 | 0.000615 |
| Sass6 | -0.83448 | 1.44E-05 | 0.000628 |
| Ypel3 | 0.646905 | 1.47E-05 | 0.000636 |
| Scamp1 | 0.567547 | 1.51E-05 | 0.000653 |
| Pkmyt1 | -0.58747 | 1.55E-05 | 0.00067 |
| Tnn | -1.07158 | 1.59E-05 | 0.000682 |
| Agpat2 | 0.844625 | 1.59E-05 | 0.000682 |
| Sgol1 | -0.6057 | 1.63E-05 | 0.000696 |
| Ccdc109b | 1.09693 | 1.65E-05 | 0.000704 |
| AABR07026424.1 | 0.720263 | 1.66E-05 | 0.000704 |
| Dctd | -0.64473 | 1.69E-05 | 0.000717 |
| LOC100910057 | 0.851294 | 1.75E-05 | 0.000735 |
| Tmem151a | 0.535175 | 1.75E-05 | 0.000735 |
| Gstt2 | 0.801708 | 1.77E-05 | 0.00074 |
| Hadhb | 0.571007 | 1.77E-05 | 0.000741 |
| Shc2 | 0.390174 | 1.79E-05 | 0.000746 |
| Mtbp | -0.51706 | 1.82E-05 | 0.000754 |
| AABR07069282.1 | -0.53709 | 1.82E-05 | 0.000754 |
| Slc16a6 | 3.193874 | 1.83E-05 | 0.000756 |
| Whsc1 | -0.51953 | 1.88E-05 | 0.000774 |
| Mastl | -0.58786 | 1.88E-05 | 0.000774 |
| Xrcc2 | -0.64355 | 1.92E-05 | 0.000788 |
| Zfp383_1 | -1.21833 | 1.94E-05 | 0.000791 |
| Cox6a2 | 1.087845 | 1.97E-05 | 0.000801 |
| Nhsl1 | -0.78688 | 2.07E-05 | 0.000842 |
| Cyp4f17 | 0.612037 | 2.09E-05 | 0.000845 |
| Por | 0.519444 | 2.18E-05 | 0.000879 |
| Fsd1 | 0.708035 | 2.19E-05 | 0.000883 |
| Ltn1 | -0.42039 | 2.32E-05 | 0.000931 |
| Ddit4 | 0.609875 | 2.35E-05 | 0.000941 |
| Atraid | 0.754796 | 2.37E-05 | 0.000944 |
| Cse1l | -0.37007 | 2.38E-05 | 0.000945 |
| Tpx2 | -0.47808 | 2.44E-05 | 0.000968 |
| Nek2 | -0.54536 | 2.47E-05 | 0.000978 |
| Rgs10 | 0.860059 | 2.48E-05 | 0.00098 |
| Asns | 0.666949 | 2.51E-05 | 0.000986 |
| Hnrnpul1 | -0.39316 | 2.51E-05 | 0.000986 |
| Nlrx1 | 0.589963 | 2.54E-05 | 0.000993 |
| Ndc80 | -0.50366 | 2.57E-05 | 0.001003 |
| Atp2a3 | 0.527562 | 2.60E-05 | 0.001011 |
| Nup85 | -0.55958 | 2.64E-05 | 0.001025 |
| Lin9 | -0.77319 | 2.74E-05 | 0.001058 |
| Dbf4 | -0.52751 | 2.79E-05 | 0.001074 |
| AABR07027407.1 | 0.583002 | 2.88E-05 | 0.001106 |
| Uchl1 | 1.075256 | 2.95E-05 | 0.001132 |
| 2-Mar | 0.635358 | 2.99E-05 | 0.001144 |
| Cdca7 | -0.78068 | 3.05E-05 | 0.001164 |
| Pfas | -0.53175 | 3.06E-05 | 0.001165 |
| AABR07054189.1 | 0.896651 | 3.19E-05 | 0.001211 |
| Rad51ap1 | -0.59689 | 3.28E-05 | 0.001241 |
| Grina | 0.525694 | 3.37E-05 | 0.001269 |
| Avpr1a | 0.348894 | 3.43E-05 | 0.001288 |
| Eef2k | 0.455884 | 3.43E-05 | 0.001288 |
| Terf1 | -1.00976 | 3.49E-05 | 0.001307 |
| Dhx9 | -0.70287 | 3.52E-05 | 0.001314 |
| Macrod1 | 0.928142 | 3.60E-05 | 0.001338 |
| Adamts15 | -0.7316 | 3.68E-05 | 0.001364 |
| Adam15 | 0.553842 | 3.72E-05 | 0.001378 |
| Amot | -0.86285 | 3.81E-05 | 0.001407 |
| Tcn2 | 0.448012 | 3.99E-05 | 0.00147 |
| Bnip3 | 0.642587 | 4.05E-05 | 0.001488 |
| Itm2b | 0.459709 | 4.11E-05 | 0.001507 |
| Ghitm | 0.574764 | 4.37E-05 | 0.001595 |
| Rad54l | -0.73573 | 4.43E-05 | 0.001613 |
| Dhrs3 | 0.520227 | 4.48E-05 | 0.001628 |
| Kif2c | -0.44026 | 4.50E-05 | 0.001631 |
| Casp8ap2 | -0.73873 | 4.58E-05 | 0.001656 |
| LOC303566 | -1.02878 | 4.66E-05 | 0.001681 |
| Exnef | -0.64259 | 4.84E-05 | 0.001742 |
| Akr1b1 | 0.737405 | 4.99E-05 | 0.00179 |
| Cenpf | -1.05083 | 5.04E-05 | 0.001802 |
| Npc2 | 0.590574 | 5.14E-05 | 0.001831 |
| Blvrb | 0.652187 | 5.14E-05 | 0.001831 |
| LOC100360726 | -0.77756 | 5.23E-05 | 0.001857 |
| Gstm1 | 0.784372 | 5.29E-05 | 0.001874 |
| Hnrpd | -0.40804 | 5.39E-05 | 0.001888 |
| Celsr2 | -0.82509 | 5.38E-05 | 0.001888 |
| Ctsd | 0.342409 | 5.37E-05 | 0.001888 |
| Slc39a1 | 0.392037 | 5.38E-05 | 0.001888 |
| Plk4 | -0.63122 | 5.43E-05 | 0.001901 |
| Sc5d | 0.390143 | 5.46E-05 | 0.001903 |
| Pold1 | -0.54073 | 5.49E-05 | 0.001909 |
| Fam46b | -0.64975 | 5.70E-05 | 0.001978 |
| Usp37 | -0.77765 | 5.87E-05 | 0.002029 |
| Mis18bp1 | -0.70886 | 5.88E-05 | 0.002029 |
| Chd3 | -0.7491 | 5.95E-05 | 0.002049 |
| Adamts7 | -0.62261 | 6.10E-05 | 0.002098 |
| Lcp1 | 0.655003 | 6.12E-05 | 0.002099 |
| Kif22 | -0.46293 | 6.14E-05 | 0.002099 |
| Psrc1 | -0.75081 | 6.18E-05 | 0.00211 |
| Gba | 0.38028 | 6.34E-05 | 0.002158 |
| Rras | 0.557998 | 6.36E-05 | 0.00216 |
| Eno2 | 0.571232 | 6.40E-05 | 0.002167 |
| Iws1 | -0.6915 | 6.42E-05 | 0.002171 |
| Ptprb | -0.77607 | 6.47E-05 | 0.002179 |
| Matn3 | -1.94072 | 6.51E-05 | 0.002189 |
| Nol3 | 0.547023 | 6.76E-05 | 0.002264 |
| Ypel5 | 0.502632 | 6.77E-05 | 0.002264 |
| Srrm1 | -0.51209 | 6.84E-05 | 0.002283 |
| Trpm4 | 0.63737 | 6.87E-05 | 0.002288 |
| Ncapg | -0.52332 | 6.89E-05 | 0.002289 |
| Hnrnpab | -0.68181 | 6.95E-05 | 0.002304 |
| Osgin1 | 1.077207 | 7.10E-05 | 0.002343 |
| Eng | 0.390725 | 7.10E-05 | 0.002343 |
| Pir | 0.570515 | 7.32E-05 | 0.002401 |
| Tspan4 | 0.67771 | 7.33E-05 | 0.002401 |
| Rttn | -0.81047 | 7.32E-05 | 0.002401 |
| Hcfc1r1 | 0.727156 | 7.40E-05 | 0.002417 |
| Notch3 | -0.70905 | 7.77E-05 | 0.002527 |
| LOC100911204 | -0.9357 | 7.77E-05 | 0.002527 |
| Tmem254 | 0.583262 | 8.13E-05 | 0.002637 |
| Smarcc1 | -0.4391 | 8.15E-05 | 0.002639 |
| Nenf | 0.659849 | 8.19E-05 | 0.002644 |
| Mmp19 | 0.350408 | 8.74E-05 | 0.002815 |
| LOC361346 | -0.50629 | 9.03E-05 | 0.002902 |
| Rft1 | 0.611404 | 9.50E-05 | 0.003046 |
| AABR07035778.2 | -1.35836 | 9.59E-05 | 0.003064 |
| Sox11 | -0.82393 | 9.60E-05 | 0.003064 |
| LOC100158225 | -0.80531 | 9.81E-05 | 0.003115 |
| Ets1 | -0.71119 | 9.78E-05 | 0.003115 |
| Ccdc88b | -0.77873 | 9.83E-05 | 0.003115 |
| Fam50a | 0.764358 | 9.88E-05 | 0.003124 |
| Hnrnpa3 | -0.5577 | 0.000101 | 0.003175 |
| Tmbim6 | 0.483812 | 0.000102 | 0.003218 |
| Slc2a4 | -0.67266 | 0.000105 | 0.003294 |
| Slc35b3 | 0.711992 | 0.000105 | 0.003301 |
| Nop58 | -0.39407 | 0.000106 | 0.003322 |
| Ifngr2 | 0.586568 | 0.000108 | 0.003365 |
| Smim20 | 0.833904 | 0.000109 | 0.003399 |
| Asb15 | -0.86837 | 0.000111 | 0.003456 |
| Psd | 0.597072 | 0.000112 | 0.003457 |
| Hadh | 0.695387 | 0.000112 | 0.003471 |
| Pik3ip1 | 0.477329 | 0.000117 | 0.0036 |
| Galk2 | 0.496394 | 0.000119 | 0.003669 |
| Lonp1 | 0.389962 | 0.000121 | 0.003718 |
| Nrm | -0.69687 | 0.000122 | 0.003718 |
| Mbnl3 | -0.87435 | 0.000122 | 0.003718 |
| Il10rb | 0.431692 | 0.000121 | 0.003718 |
| Lsp1 | -0.41373 | 0.000123 | 0.003732 |
| Acss2 | 0.526968 | 0.000124 | 0.003755 |
| Vti1b | 0.726615 | 0.000124 | 0.003761 |
| Tbc1d9b | 0.407979 | 0.000127 | 0.003839 |
| Atf4 | 0.43867 | 0.000127 | 0.003839 |
| Ewsr1 | -0.62014 | 0.000131 | 0.00393 |
| Fam173a | 0.668004 | 0.000131 | 0.00393 |
| E2f1 | -0.49461 | 0.000131 | 0.003937 |
| Palb2 | -0.65739 | 0.000133 | 0.003969 |
| Mcoln1 | 0.409455 | 0.000133 | 0.003981 |
| Tfdp1 | -0.43501 | 0.000134 | 0.00399 |
| Gpr108 | 0.363291 | 0.000135 | 0.00402 |
| Melk | -0.63919 | 0.000136 | 0.004027 |
| Lxn | 0.582573 | 0.000136 | 0.004032 |
| Nup155 | -0.41399 | 0.000138 | 0.004065 |
| Nradd | 0.704716 | 0.000139 | 0.004109 |
| Tardbp | -0.51882 | 0.000141 | 0.004141 |
| Nuf2 | -0.48336 | 0.000142 | 0.004182 |
| Arhgef39 | -0.68806 | 0.000146 | 0.004287 |
| Sec14l2 | 0.847316 | 0.000151 | 0.004407 |
| Abcc4 | 0.484666 | 0.000151 | 0.004417 |
| Map1lc3a | 0.712952 | 0.000152 | 0.004419 |
| Plekhg3 | -0.71698 | 0.000153 | 0.004444 |
| Npdc1 | 0.524407 | 0.000153 | 0.004444 |
| Creb3l1 | 0.310809 | 0.000156 | 0.004506 |
| Fancb | -0.79472 | 0.000156 | 0.004507 |
| Mum1l1 | -0.51062 | 0.000157 | 0.004531 |
| Ighm | -0.75401 | 0.000158 | 0.004541 |
| Atp13a2 | 0.431849 | 0.00016 | 0.004589 |
| Trpv2 | 0.831282 | 0.000162 | 0.004635 |
| Epb41l2 | -0.58332 | 0.000162 | 0.004642 |
| Ramp2 | 0.791098 | 0.000163 | 0.004642 |
| Slc7a5 | 0.315282 | 0.000164 | 0.004681 |
| Krt80 | -0.6698 | 0.000167 | 0.004749 |
| Cenpe | -0.94176 | 0.000173 | 0.004892 |
| Pbk | -0.63166 | 0.000174 | 0.004911 |
| Ano10 | 0.462172 | 0.000176 | 0.004959 |
| Hid1 | 0.524709 | 0.000177 | 0.005001 |
| AABR07051550.1 | 0.968405 | 0.000178 | 0.005006 |
| Crybg3 | -0.67839 | 0.00018 | 0.005038 |
| Fuca1 | 0.550446 | 0.00018 | 0.005038 |
| Pla2g6 | 0.496993 | 0.000181 | 0.005048 |
| Plxnb1 | -0.80474 | 0.000183 | 0.005101 |
| Lgmn | 0.332528 | 0.000186 | 0.005175 |
| Stard10 | 1.02583 | 0.000193 | 0.005375 |
| Nfe2l2 | 0.326017 | 0.000195 | 0.005396 |
| Tmbim1 | 0.442897 | 0.000197 | 0.005457 |
| Pfn2 | 0.476712 | 0.000199 | 0.00548 |
| Cenpj | -0.84757 | 0.000199 | 0.00548 |
| Mmp11 | 0.447934 | 0.000199 | 0.00548 |
| Kctd15 | 0.356114 | 0.000205 | 0.005615 |
| Pim1 | -0.79827 | 0.000206 | 0.005635 |
| Zgrf1 | -0.71008 | 0.00021 | 0.005745 |
| Ckap2 | -0.41223 | 0.000211 | 0.00577 |
| Prr7 | 0.687837 | 0.000213 | 0.005792 |
| Tyro3 | 0.619607 | 0.000213 | 0.005792 |
| Slc1a5 | 0.402394 | 0.000214 | 0.005813 |
| Hspb8 | 0.578885 | 0.000217 | 0.005879 |
| Tra2b | -0.32622 | 0.000219 | 0.005905 |
| Fcgrt | 0.494103 | 0.000219 | 0.005905 |
| Itm2c | 0.416406 | 0.000221 | 0.005955 |
| RGD1304694 | 0.406265 | 0.000222 | 0.005955 |
| Hadha | 0.460268 | 0.000224 | 0.006019 |
| Pln | -0.86408 | 0.000229 | 0.006121 |
| Lin54 | -0.47272 | 0.000229 | 0.006125 |
| Pcdh18 | -0.57367 | 0.000233 | 0.00619 |
| Acta1 | -0.62417 | 0.000233 | 0.00619 |
| Rab31 | 0.31449 | 0.000233 | 0.00619 |
| Sfpq | -0.57247 | 0.000232 | 0.00619 |
| Smc1a | -0.56519 | 0.000237 | 0.006273 |
| Sigmar1 | 0.637261 | 0.000238 | 0.006288 |
| Slc25a20 | 0.713303 | 0.000239 | 0.006305 |
| LOC688459 | 0.618689 | 0.00024 | 0.006305 |
| Ift46 | 0.508773 | 0.000243 | 0.006364 |
| Rif1 | -0.80117 | 0.000243 | 0.006364 |
| LOC102556347 | 0.483627 | 0.000244 | 0.006382 |
| Spsb2 | 0.484879 | 0.000245 | 0.006404 |
| Senp1 | -0.55329 | 0.000247 | 0.006439 |
| Prss23 | -0.46394 | 0.000251 | 0.006523 |
| Rgs17 | 0.724562 | 0.000257 | 0.006665 |
| Sesn3 | 0.473245 | 0.000258 | 0.006684 |
| Inpp5k | 0.585938 | 0.000258 | 0.006684 |
| Tex264 | 0.546649 | 0.000259 | 0.00669 |
| Polh | -0.47254 | 0.000261 | 0.006732 |
| Gdpd1 | 0.582869 | 0.000263 | 0.006762 |
| Desi1 | 0.504265 | 0.000266 | 0.006824 |
| Cnot6 | -0.64661 | 0.000268 | 0.006861 |
| Cenpo | -0.559 | 0.000269 | 0.006891 |
| Gss | 0.667221 | 0.000271 | 0.006921 |
| Spc24 | -0.58314 | 0.000272 | 0.006925 |
| Nolc1 | -0.29428 | 0.000273 | 0.00694 |
| Mib2 | 0.375499 | 0.000275 | 0.006994 |
| Bcat2 | 0.587515 | 0.000277 | 0.007011 |
| LOC100911453 | -7.70498 | 0.000278 | 0.007039 |
| Khdrbs1 | -0.32715 | 0.000279 | 0.007056 |
| Usp1 | -0.55279 | 0.000281 | 0.007081 |
| Sipa1 | -0.40416 | 0.000292 | 0.007338 |
| Btbd6 | 0.484421 | 0.000293 | 0.00735 |
| Cd59 | 0.553419 | 0.000293 | 0.007356 |
| Bambi | 0.574144 | 0.000298 | 0.007449 |
| Cntrl | -0.72986 | 0.000299 | 0.007466 |
| Hm13 | 0.564662 | 0.0003 | 0.007495 |
| Taf15 | -0.62528 | 0.000303 | 0.007534 |
| Itpk1 | 0.678485 | 0.000303 | 0.007536 |
| Hrasls | 0.546057 | 0.000305 | 0.007558 |
| Smarca5 | -0.53441 | 0.000307 | 0.007599 |
| Tnfrsf1a | 0.30162 | 0.000307 | 0.007599 |
| Gstt1 | 1.10614 | 0.000316 | 0.007796 |
| Prss12 | 0.612949 | 0.000319 | 0.007849 |
| LOC100911625 | 0.599346 | 0.000319 | 0.007849 |
| Rhbdl3 | 0.793696 | 0.000323 | 0.007929 |
| RGD1306227 | -0.68528 | 0.000324 | 0.007929 |
| RGD1565033 | 0.56459 | 0.000325 | 0.007956 |
| Fam69b | 0.607229 | 0.000328 | 0.00802 |
| Ptdss1 | 0.458418 | 0.000342 | 0.008333 |
| Zbtb20 | -1.26621 | 0.000345 | 0.008363 |
| Slc43a2 | 0.531941 | 0.000345 | 0.008363 |
| Irf5 | 0.608994 | 0.000346 | 0.008363 |
| Aars | 0.374949 | 0.000344 | 0.008363 |
| LOC100911615 | 0.420993 | 0.000346 | 0.008363 |
| Emp1 | -0.3267 | 0.00035 | 0.008443 |
| Kcnd3 | -0.8283 | 0.000351 | 0.00845 |
| Cntrob | -0.67367 | 0.000352 | 0.008471 |
| Slc29a3 | 0.407653 | 0.000355 | 0.00851 |
| Dirc2 | 0.409606 | 0.000355 | 0.00851 |
| Txnrd3 | 0.597971 | 0.000359 | 0.008574 |
| AABR07002623.2 | 0.707779 | 0.000366 | 0.008728 |
| LOC100910255 | -0.63028 | 0.000368 | 0.008779 |
| Nedd1 | -0.47886 | 0.000373 | 0.008879 |
| Tmem150a | 0.443382 | 0.000374 | 0.008882 |
| Enpp1 | 0.355407 | 0.000378 | 0.008942 |
| RGD1559896 | -0.30763 | 0.000377 | 0.008942 |
| Rasl11b | 0.423302 | 0.000382 | 0.009018 |
| Rgs2 | 0.710488 | 0.000384 | 0.009056 |
| Atpaf2 | 0.637028 | 0.000385 | 0.009056 |
| Faxdc2 | 1.215105 | 0.000387 | 0.009098 |
| Plagl1 | -0.93337 | 0.000393 | 0.009223 |
| Anxa7 | 0.500234 | 0.000395 | 0.009256 |
| Cxcl12 | -0.43042 | 0.000396 | 0.009273 |
| Ccdc18 | -0.85635 | 0.000402 | 0.009383 |
| As3mt | 0.591655 | 0.000403 | 0.009406 |
| Parp3 | 0.475146 | 0.000418 | 0.009731 |
| LOC100911293 | 0.564088 | 0.000419 | 0.009739 |
| Lsm6 | -1.05803 | 0.000421 | 0.009761 |
| Tmem160 | 0.901001 | 0.000422 | 0.009761 |
| Tspan31 | 0.624538 | 0.000422 | 0.009761 |
| AW549877 | -0.67108 | 0.000423 | 0.00977 |
| Kif18a | -0.69055 | 0.000438 | 0.010095 |
| Casq2 | -0.31175 | 0.000442 | 0.010164 |
| Klf11 | 0.578935 | 0.000451 | 0.010358 |
| Tmem115 | 0.408714 | 0.000453 | 0.01039 |
| Mvd | 0.563694 | 0.000464 | 0.010618 |
| Txlna | -0.36867 | 0.000465 | 0.010618 |
| Rrm2 | -0.49032 | 0.000467 | 0.010646 |
| Pole2 | -0.76317 | 0.000468 | 0.010668 |
| Ckap5 | -0.43586 | 0.00047 | 0.010683 |
| AABR07026359.1 | -1.10034 | 0.000485 | 0.011003 |
| Ccdc36 | -1.18709 | 0.000488 | 0.011064 |
| Cd81 | 0.336435 | 0.000489 | 0.011071 |
| Polr3b | -0.34593 | 0.000492 | 0.011109 |
| Smc3 | -0.50662 | 0.000493 | 0.011127 |
| Agtrap | 0.472269 | 0.000507 | 0.011407 |
| LOC100912399 | -0.88625 | 0.000507 | 0.011407 |
| Tmem256 | 0.725211 | 0.000525 | 0.011791 |
| Serpinb6 | 0.586701 | 0.000526 | 0.011798 |
| Ankrd32 | -0.50977 | 0.000529 | 0.011836 |
| Brd8 | -0.42195 | 0.000531 | 0.011857 |
| Fkbp5 | -0.49895 | 0.000535 | 0.011924 |
| Mybph | -1.87364 | 0.000537 | 0.011945 |
| Pkm | 0.696875 | 0.000536 | 0.011945 |
| Tap2 | 0.787042 | 0.000551 | 0.012224 |
| Rb1 | -0.64707 | 0.000556 | 0.012314 |
| Stmn3 | 0.843015 | 0.000557 | 0.012318 |
| Prim2 | -0.57079 | 0.000563 | 0.012437 |
| Bid | 0.42565 | 0.000571 | 0.012589 |
| Hnrnpm | -0.64987 | 0.000579 | 0.012693 |
| Hnrnpf | -0.32448 | 0.00058 | 0.012693 |
| Sh3glb2 | 0.614767 | 0.000579 | 0.012693 |
| Egfl7 | 0.620172 | 0.00058 | 0.012693 |
| Dbi | 0.571072 | 0.000579 | 0.012693 |
| Otub2 | 0.451316 | 0.000589 | 0.012877 |
| Cd47 | 0.333757 | 0.000591 | 0.012894 |
| Tmem180 | 0.621035 | 0.000599 | 0.013042 |
| Abhd14b | 0.570659 | 0.000604 | 0.013132 |
| Ppp2r5b | 0.407973 | 0.000605 | 0.013136 |
| Rn60_6_1241.2 | -0.60768 | 0.000615 | 0.013338 |
| Kras | -0.54673 | 0.000616 | 0.013342 |
| Etv5 | 0.343713 | 0.000622 | 0.013436 |
| Skp2 | -0.45832 | 0.000627 | 0.013528 |
| Ctsb | 0.297393 | 0.000631 | 0.013581 |
| Tmem223 | 0.613562 | 0.000631 | 0.013581 |
| Nrp1 | -0.64863 | 0.000635 | 0.013636 |
| Tmem147 | 0.675832 | 0.000639 | 0.013682 |
| RGD1561161 | -0.61231 | 0.000639 | 0.013682 |
| Ppat | -0.48829 | 0.000648 | 0.013827 |
| Pprc1 | -0.42912 | 0.000647 | 0.013827 |
| Acot7 | 0.60304 | 0.000649 | 0.013837 |
| Ldlr | 0.429665 | 0.000652 | 0.013866 |
| Fam189b | 0.375019 | 0.000676 | 0.014358 |
| Mb21d1 | -0.8423 | 0.000678 | 0.014385 |
| Wdr76 | -0.72598 | 0.000682 | 0.01442 |
| Tfec | 0.619562 | 0.000681 | 0.01442 |
| MGC105649 | 1.035502 | 0.000688 | 0.014529 |
| Atp10a | -0.56806 | 0.000691 | 0.014576 |
| C2cd3 | -0.54963 | 0.000695 | 0.01461 |
| Stat2 | 0.34146 | 0.000694 | 0.01461 |
| AABR07047844.1 | -3.45142 | 0.0007 | 0.014696 |
| Prss35 | -0.53036 | 0.000702 | 0.014723 |
| Pkm | 0.584366 | 0.000706 | 0.014753 |
| Ska3 | -0.53341 | 0.000705 | 0.014753 |
| Prdx1 | 0.441793 | 0.000711 | 0.014836 |
| Glo1 | 0.557448 | 0.00073 | 0.015197 |
| Amer1 | -0.40658 | 0.000732 | 0.015197 |
| Diaph3 | -0.46071 | 0.000731 | 0.015197 |
| Mtr | -0.53216 | 0.000735 | 0.015248 |
| Fign | -0.91662 | 0.000739 | 0.015255 |
| Cdkn2c | -0.46142 | 0.00074 | 0.015255 |
| Dot1l | -0.67966 | 0.000739 | 0.015255 |
| Mns1 | -0.84607 | 0.000739 | 0.015255 |
| Sart3 | -0.34002 | 0.00075 | 0.015354 |
| Aldh2 | 0.508825 | 0.000747 | 0.015354 |
| Tbc1d31 | -0.4199 | 0.00075 | 0.015354 |
| Rab4a | 0.602914 | 0.000749 | 0.015354 |
| Cers2 | 0.329822 | 0.00075 | 0.015354 |
| Nup205 | -0.3404 | 0.000753 | 0.015397 |
| Cenpw | -0.49224 | 0.000762 | 0.01555 |
| Cenpc | -0.48399 | 0.000764 | 0.015561 |
| Camk1 | 0.586682 | 0.000767 | 0.015599 |
| Nexn | -0.58669 | 0.000778 | 0.015807 |
| Acat2 | 0.646331 | 0.0008 | 0.01621 |
| Sipa1l2 | -0.50295 | 0.0008 | 0.01621 |
| LOC691422 | -0.62311 | 0.000803 | 0.01624 |
| Rn60_10_0698.6 | 0.966756 | 0.000807 | 0.016299 |
| Tspo | 0.657938 | 0.000811 | 0.016354 |
| Myh11 | -0.70776 | 0.000813 | 0.016384 |
| LOC103689982 | 0.322888 | 0.000816 | 0.016412 |
| Rab3d | 0.424475 | 0.000818 | 0.016431 |
| Cep250 | -1.00901 | 0.000821 | 0.016467 |
| Cep135 | -0.65303 | 0.000823 | 0.016479 |
| Gper1 | -0.87282 | 0.000824 | 0.016482 |
| Nfyc | -0.44068 | 0.00083 | 0.016574 |
| Psmd11 | 0.441411 | 0.000833 | 0.016616 |
| Ccdc64b | -2.22186 | 0.000837 | 0.016652 |
| Trim59 | -0.38015 | 0.000837 | 0.016652 |
| Crat | 0.475768 | 0.000844 | 0.016736 |
| Ncl | -0.355 | 0.000844 | 0.016736 |
| Dtnb | 0.589399 | 0.000862 | 0.017039 |
| Gys1 | 0.370811 | 0.000861 | 0.017039 |
| Uqcr10 | 0.780116 | 0.000867 | 0.017112 |
| Parp10 | 0.63507 | 0.000871 | 0.017165 |
| Mvk | 0.612744 | 0.000875 | 0.01722 |
| Nudt8 | 0.81477 | 0.000878 | 0.017266 |
| Arl6ip6 | -0.53983 | 0.000883 | 0.017313 |
| Naa40 | -0.5073 | 0.000882 | 0.017313 |
| Snta1 | 0.560291 | 0.000889 | 0.017407 |
| Aplp1 | 0.367556 | 0.000898 | 0.017551 |
| RGD1562037 | -0.51749 | 0.000899 | 0.017551 |
| Phgdh | 0.561014 | 0.000903 | 0.017605 |
| Rbm12 | -1.20047 | 0.000914 | 0.017789 |
| Adora1 | 0.469409 | 0.000923 | 0.017945 |
| Hmgb2l1 | -0.85596 | 0.000931 | 0.018051 |
| AABR07052758.1 | 0.47177 | 0.00093 | 0.018051 |
| Ssx2ip | -0.36462 | 0.000934 | 0.018078 |
| Ndfip1 | 0.376414 | 0.000938 | 0.018136 |
| Chtf18 | -0.58299 | 0.000941 | 0.018165 |
| Mgmt | 0.603168 | 0.000945 | 0.018221 |
| Jtb | 0.394531 | 0.00095 | 0.01829 |
| Dnpep | 0.53895 | 0.000955 | 0.018363 |
| Ppp1r15a | 0.355318 | 0.000984 | 0.018888 |
| Bora | -0.58424 | 0.000992 | 0.019011 |
| Slc9a9 | 0.673393 | 0.001002 | 0.019185 |
| Prrx2 | 0.468246 | 0.001022 | 0.019545 |
| Nrcam | 1.303447 | 0.001024 | 0.019557 |
| Aunip | -0.8643 | 0.001034 | 0.019706 |
| Hnrnph2 | -0.39086 | 0.001036 | 0.019709 |
| Ifitm3 | 0.598431 | 0.001036 | 0.019709 |
| Bri3bp | 0.480417 | 0.001039 | 0.019709 |
| Mthfd1 | -0.37457 | 0.001041 | 0.019709 |
| Dpep1 | 0.468969 | 0.001041 | 0.019709 |
| Wdr62 | -0.32272 | 0.001043 | 0.019727 |
| Shc1 | 0.276854 | 0.001051 | 0.019847 |
| Rbm28 | -0.33741 | 0.001055 | 0.019903 |
| Ahi1 | 0.386074 | 0.001057 | 0.019903 |
| Otud3 | -0.56707 | 0.001058 | 0.019903 |
| Ago2 | -0.63249 | 0.001061 | 0.019921 |
| Hax1 | 0.614328 | 0.001064 | 0.01995 |
| Pabpn1 | -0.38704 | 0.001068 | 0.020005 |
| Ptgs2 | -0.62336 | 0.001072 | 0.020033 |
| LOC360919 | 0.702664 | 0.001071 | 0.020033 |
| Sltm | -0.57095 | 0.001075 | 0.020065 |
| Gpt2 | 0.289302 | 0.001082 | 0.020162 |
| Nit2 | 0.728485 | 0.001085 | 0.020187 |
| Hsd17b10 | 0.692459 | 0.001089 | 0.020205 |
| Ltbr | 0.337649 | 0.001087 | 0.020205 |
| Nup153 | -0.6161 | 0.001091 | 0.020222 |
| Fmr1 | -0.70385 | 0.001098 | 0.020327 |
| Stac2 | 0.678126 | 0.001105 | 0.020419 |
| Hnrnpa2b1 | -0.46639 | 0.001109 | 0.020466 |
| Tecr | 0.607237 | 0.001112 | 0.020502 |
| Kifc1 | -0.43297 | 0.001123 | 0.020642 |
| LOC100910429 | -0.60198 | 0.001121 | 0.020642 |
| Vwa5a | 0.354589 | 0.001126 | 0.020672 |
| Zfp84 | -0.71762 | 0.001138 | 0.020863 |
| Cpq | 0.281714 | 0.001141 | 0.020876 |
| Rnf166 | 0.435867 | 0.001141 | 0.020876 |
| Spon2 | 0.335026 | 0.001145 | 0.020887 |
| Cdc25b | -0.30652 | 0.001144 | 0.020887 |
| Mis18a | -0.66075 | 0.001152 | 0.02098 |
| Dnajc21 | -0.5922 | 0.001156 | 0.021042 |
| Mif4gd | 0.574663 | 0.001167 | 0.021211 |
| Gli2 | -0.58866 | 0.001174 | 0.021277 |
| Plxnd1 | -0.45188 | 0.001173 | 0.021277 |
| Gabarapl1 | 0.512094 | 0.001176 | 0.021282 |
| Med14 | -0.58719 | 0.001187 | 0.021426 |
| Smc6 | -0.42078 | 0.001187 | 0.021426 |
| Rc3h1 | -0.59106 | 0.00119 | 0.021453 |
| LOC100912481 | -0.69953 | 0.001192 | 0.021458 |
| Slc46a1 | 0.603739 | 0.001194 | 0.021476 |
| Ocm2 | 0.850685 | 0.001203 | 0.021572 |
| Grhpr | 0.723085 | 0.001203 | 0.021572 |
| Trappc6a | 0.64957 | 0.001211 | 0.021648 |
| Wdr90 | -0.49079 | 0.001211 | 0.021648 |
| Palm2 | -0.73999 | 0.001212 | 0.021648 |
| Tmem50a | 0.464324 | 0.001227 | 0.02189 |
| Atm | -0.62779 | 0.001229 | 0.0219 |
| Apaf1 | -0.62226 | 0.001232 | 0.021921 |
| Zwilch | -0.39619 | 0.001234 | 0.021942 |
| Slc35e4 | 0.362351 | 0.001237 | 0.021952 |
| Mbd4 | -0.85559 | 0.001238 | 0.021952 |
| Sars | 0.530134 | 0.001241 | 0.02198 |
| LOC100911713 | 0.747624 | 0.001244 | 0.021994 |
| Pds5b | -0.51822 | 0.001255 | 0.022048 |
| Paxip1 | -0.73974 | 0.001254 | 0.022048 |
| Gpam | -0.40529 | 0.001254 | 0.022048 |
| Leprot | 0.361341 | 0.00125 | 0.022048 |
| Card9 | 0.363625 | 0.001252 | 0.022048 |
| Arpc5l | 0.507521 | 0.001266 | 0.022224 |
| Zscan22 | -0.58609 | 0.001269 | 0.022237 |
| Arhgap33 | -1.05833 | 0.001271 | 0.022261 |
| Smchd1 | -0.56711 | 0.001276 | 0.022312 |
| Timp3 | -0.45101 | 0.001279 | 0.022338 |
| Pi4kb | 0.317444 | 0.001285 | 0.022414 |
| N4bp3 | -0.69552 | 0.001295 | 0.022536 |
| Cdc45 | -0.50146 | 0.001294 | 0.022536 |
| Adh7 | 0.773058 | 0.001314 | 0.022798 |
| Lima1 | -0.36437 | 0.001313 | 0.022798 |
| Pigx | 0.598838 | 0.001327 | 0.023006 |
| Aurkb | -0.37245 | 0.001334 | 0.023065 |
| Mea1 | 0.71808 | 0.001335 | 0.023065 |
| Tbc1d17 | 0.357691 | 0.001336 | 0.023065 |
| Hint2 | 0.802696 | 0.001338 | 0.023082 |
| Rbm25 | -0.6702 | 0.001347 | 0.02319 |
| Csrp2 | 0.481865 | 0.001348 | 0.02319 |
| B4galt4 | 1.074559 | 0.001351 | 0.023217 |
| Pcbd2 | 0.777202 | 0.001359 | 0.02333 |
| Zfp383 | 1.126696 | 0.001363 | 0.023371 |
| Rngtt | -0.41454 | 0.001374 | 0.023532 |
| Mylip | -0.5861 | 0.001382 | 0.023635 |
| LOC100911730 | 0.380308 | 0.001391 | 0.023749 |
| Commd4 | 0.504589 | 0.001395 | 0.023797 |
| Cnnm2 | -0.42354 | 0.001398 | 0.023797 |
| AC139950.1 | -0.40908 | 0.001399 | 0.023797 |
| Uckl1 | 0.36709 | 0.001406 | 0.023899 |
| Fbxo6 | 0.58288 | 0.00141 | 0.023928 |
| Akr7a2 | 0.64038 | 0.001413 | 0.02396 |
| Chchd6 | 0.72362 | 0.001426 | 0.024145 |
| Fez1 | 1.287746 | 0.001437 | 0.02431 |
| Pld3 | 0.28421 | 0.001444 | 0.0244 |
| Ccdc110 | 2.080559 | 0.001455 | 0.024545 |
| Snrnp200 | -0.27925 | 0.001459 | 0.024593 |
| Slc7a7 | 0.60893 | 0.00147 | 0.024711 |
| Htatsf1 | -0.55093 | 0.001468 | 0.024711 |
| Btc | 0.951918 | 0.001473 | 0.02474 |
| Mybl2 | -0.43488 | 0.001477 | 0.024761 |
| Pom121 | -0.38625 | 0.001496 | 0.025055 |
| Itgb5 | 0.250457 | 0.0015 | 0.025095 |
| Cdkl5 | -0.75113 | 0.001504 | 0.025095 |
| Cep85 | -0.52946 | 0.001503 | 0.025095 |
| Tm6sf1 | 0.455994 | 0.001507 | 0.025115 |
| Hddc3 | 0.597331 | 0.001517 | 0.025262 |
| Maf1 | 0.553748 | 0.001529 | 0.025409 |
| RGD1359290 | 0.818926 | 0.00153 | 0.025409 |
| Nfic | -0.32966 | 0.001534 | 0.025421 |
| Dctn3 | 0.605074 | 0.001536 | 0.025421 |
| Kif5b | -0.44841 | 0.001535 | 0.025421 |
| Pbrm1 | -0.62795 | 0.001541 | 0.02547 |
| Derl2 | 0.661717 | 0.001548 | 0.025567 |
| Atp6v0b | 0.531131 | 0.001559 | 0.025717 |
| Tk1 | -0.46561 | 0.001563 | 0.025745 |
| Islr | 0.300388 | 0.001578 | 0.025956 |
| Wbp11 | -0.40496 | 0.001582 | 0.025992 |
| Aprt | 0.57004 | 0.001596 | 0.026205 |
| Chaf1b | -0.52158 | 0.001599 | 0.026213 |
| Dok4 | 0.577208 | 0.001606 | 0.026292 |
| Cox7a2 | 1.538807 | 0.00162 | 0.026492 |
| Ttc39c | 0.410297 | 0.001628 | 0.026604 |
| Abcb6 | 0.455691 | 0.001633 | 0.026609 |
| RGD1563815 | 0.577117 | 0.001631 | 0.026609 |
| Chst3 | -0.81123 | 0.001641 | 0.026716 |
| Tceal5 | -0.45232 | 0.001644 | 0.026729 |
| Gzmb | 1.167148 | 0.001647 | 0.026757 |
| Hk2 | -0.61714 | 0.001651 | 0.026781 |
| Pigy | 0.475568 | 0.001653 | 0.026781 |
| Pif1 | -0.80267 | 0.001658 | 0.026829 |
| Cyp27a1 | 0.771765 | 0.001666 | 0.02693 |
| Srsf4 | -0.32035 | 0.00167 | 0.026944 |
| Zfp275 | -0.60475 | 0.001671 | 0.026944 |
| Me1 | 0.311255 | 0.001677 | 0.027019 |
| Cand1 | -0.25238 | 0.00168 | 0.02704 |
| Myc | 0.399561 | 0.001691 | 0.027178 |
| Rn60_14_0846.1 | -0.40861 | 0.001694 | 0.027197 |
| Hltf | -0.43869 | 0.001702 | 0.027288 |
| Npat | -0.43924 | 0.001706 | 0.027318 |
| Rasgrp3 | -0.58964 | 0.001712 | 0.027382 |
| Safb | -0.46232 | 0.001716 | 0.027417 |
| Hist1h2bg | 0.638808 | 0.001728 | 0.027584 |
| Phf6 | -0.40393 | 0.001744 | 0.027651 |
| Cep295 | -0.66446 | 0.001744 | 0.027651 |
| Psip1 | -0.40532 | 0.001736 | 0.027651 |
| Cnot1 | -0.45732 | 0.001738 | 0.027651 |
| Clptm1 | 0.331934 | 0.001738 | 0.027651 |
| Crlf2 | 0.636437 | 0.001742 | 0.027651 |
| Proser3 | -0.48073 | 0.001749 | 0.02769 |
| Aox1 | 0.610227 | 0.001756 | 0.027779 |
| Cib2 | 0.725271 | 0.001761 | 0.027818 |
| RGD1307554 | -0.30481 | 0.001766 | 0.02787 |
| Hoxd9 | 0.460131 | 0.00177 | 0.02789 |
| Tmem185a | 0.35446 | 0.001771 | 0.02789 |
| Smc5 | -0.46588 | 0.001778 | 0.027957 |
| Zranb3 | -0.5123 | 0.001788 | 0.028084 |
| Tbc1d15 | -0.29669 | 0.001791 | 0.028097 |
| Rab11b | 0.460036 | 0.001796 | 0.028155 |
| Snf8 | 0.517627 | 0.001817 | 0.028443 |
| Hebp1 | 0.37043 | 0.001823 | 0.028502 |
| Rps6ka5 | -0.55975 | 0.001825 | 0.028502 |
| Prx | -0.62181 | 0.001831 | 0.028548 |
| Tnfaip8 | 0.465432 | 0.001832 | 0.028548 |
| Topors | -0.45777 | 0.001844 | 0.028708 |
| Ube2e2 | 0.803347 | 0.001848 | 0.028745 |
| Znhit2 | 0.619918 | 0.001856 | 0.028826 |
| Steap1 | 0.586356 | 0.001863 | 0.028879 |
| Tmem128 | 0.499167 | 0.001863 | 0.028879 |
| Gdf1 | 0.621898 | 0.001867 | 0.028914 |
| Laptm4b | 0.402082 | 0.001874 | 0.02898 |
| AABR07065886.1 | -0.49966 | 0.001878 | 0.029017 |
| Baz1b | -0.53743 | 0.001893 | 0.02914 |
| Ephx1 | 0.627418 | 0.001891 | 0.02914 |
| Ttk | -0.44996 | 0.001892 | 0.02914 |
| Dnajb5 | -0.28424 | 0.001897 | 0.02918 |
| Rab34 | 0.403042 | 0.0019 | 0.029186 |
| Ubr7 | -0.31166 | 0.001903 | 0.0292 |
| Daxx | -0.33011 | 0.001907 | 0.029233 |
| Cpa6 | 0.803549 | 0.001909 | 0.029233 |
| Tubgcp6 | -0.31357 | 0.001912 | 0.029238 |
| RT1-T24-1 | -0.74392 | 0.001914 | 0.029238 |
| Capg | 0.600886 | 0.001922 | 0.029337 |
| Naa15 | -0.51216 | 0.001932 | 0.029461 |
| Atp13a3 | -0.81212 | 0.001935 | 0.029468 |
| Mat1a | 0.653577 | 0.001938 | 0.029483 |
| Ano5 | -1.35151 | 0.001953 | 0.029545 |
| Rnf6 | -0.70499 | 0.001951 | 0.029545 |
| Tfrc | -0.397 | 0.001949 | 0.029545 |
| Tns1 | -0.50349 | 0.001952 | 0.029545 |
| Carkd | 0.596827 | 0.001953 | 0.029545 |
| Serpine2 | 0.31313 | 0.001959 | 0.029605 |
| Hcn2 | 0.509456 | 0.001995 | 0.030112 |
| Ctps1 | -0.4249 | 0.002002 | 0.030195 |
| Ifnar1 | 0.281115 | 0.002007 | 0.030237 |
| Josd2 | 0.548809 | 0.002013 | 0.030259 |
| B3gat3 | 0.47597 | 0.002011 | 0.030259 |
| Pcyox1l | -0.48767 | 0.002022 | 0.030362 |
| Slc37a3 | 0.299548 | 0.002043 | 0.030637 |
| Aldh5a1 | 1.234825 | 0.002054 | 0.030758 |
| Spcs1 | 0.435835 | 0.002055 | 0.030758 |
| Serbp1 | -0.24871 | 0.002063 | 0.030803 |
| Slc39a10 | -0.51293 | 0.002061 | 0.030803 |
| Mad2l1 | -0.39019 | 0.00207 | 0.030851 |
| Ankrd26 | -0.72111 | 0.002072 | 0.030851 |
| Dclre1b | -0.41848 | 0.002071 | 0.030851 |
| Lnp | -0.53891 | 0.002081 | 0.030953 |
| Stat5a | 0.493397 | 0.002093 | 0.03109 |
| Glmp | 0.33299 | 0.002109 | 0.031294 |
| RGD1561113 | 0.984723 | 0.002115 | 0.031315 |
| Orc6 | -0.59121 | 0.002115 | 0.031315 |
| Mcmbp | -0.30056 | 0.00212 | 0.03136 |
| Emc3 | 0.368838 | 0.002129 | 0.031423 |
| Narfl | 0.476292 | 0.002129 | 0.031423 |
| Net1 | -0.75364 | 0.002133 | 0.031446 |
| Slc6a7 | -1.08183 | 0.002137 | 0.031482 |
| Mex3a | -0.69401 | 0.002143 | 0.031524 |
| Eif4ebp1 | 0.45121 | 0.002151 | 0.031609 |
| Bnip3l | 0.354029 | 0.002155 | 0.031637 |
| Mt2A | 2.077371 | 0.002179 | 0.03194 |
| Ngfrap1 | 0.462201 | 0.00218 | 0.03194 |
| Use1 | 0.70172 | 0.002194 | 0.032114 |
| Spns1 | 0.353636 | 0.002205 | 0.032246 |
| Slc27a1 | 0.338294 | 0.002212 | 0.032279 |
| Taf5 | -0.54882 | 0.002211 | 0.032279 |
| Fam178a | -0.54306 | 0.002223 | 0.032393 |
| LOC100364190 | -1.51287 | 0.00223 | 0.032436 |
| Zc3h18 | -0.34252 | 0.002229 | 0.032436 |
| Hrc | -1.36471 | 0.002258 | 0.032769 |
| Pnn | -0.85284 | 0.002258 | 0.032769 |
| Ptgs1 | 0.552205 | 0.002265 | 0.032841 |
| Renbp | 0.444488 | 0.002267 | 0.032841 |
| Dck | -0.5514 | 0.002276 | 0.032874 |
| Tmem181 | 0.319955 | 0.002275 | 0.032874 |
| Zdhhc24 | 0.473361 | 0.002278 | 0.032874 |
| Sf1 | -0.25478 | 0.002279 | 0.032874 |
| Arhgef26 | -0.71896 | 0.002304 | 0.033197 |
| Cldn15 | -0.80532 | 0.00231 | 0.033212 |
| Alg2 | 0.345052 | 0.00231 | 0.033212 |
| Rn60_20_0056.5 | -1.00598 | 0.002316 | 0.033239 |
| Sgca | 0.345942 | 0.002316 | 0.033239 |
| Anxa4 | 0.344268 | 0.002326 | 0.033305 |
| Pck2 | 0.54122 | 0.002324 | 0.033305 |
| Asf1b | -0.47191 | 0.002357 | 0.033647 |
| Cyba | 0.645467 | 0.002355 | 0.033647 |
| Sin3a | -0.35968 | 0.002356 | 0.033647 |
| Urb1 | -0.53427 | 0.002386 | 0.034021 |
| Hspa2 | -0.56834 | 0.002401 | 0.034205 |
| Mrps33 | 0.604594 | 0.002406 | 0.034241 |
| RGD1307752 | 0.62414 | 0.002414 | 0.034316 |
| Slc38a7 | 0.284459 | 0.002418 | 0.034347 |
| Smad5 | -0.4 | 0.002427 | 0.034432 |
| Ccne1 | -0.4014 | 0.002437 | 0.034511 |
| Peak1 | -0.67887 | 0.002437 | 0.034511 |
| Hn1l | -0.34412 | 0.002448 | 0.034625 |
| Angel1 | 0.492604 | 0.002469 | 0.034884 |
| Supt16h | -0.38083 | 0.002475 | 0.034902 |
| Hsd17b4 | 0.367345 | 0.002474 | 0.034902 |
| Ccdc15 | -0.93248 | 0.002484 | 0.034992 |
| Plekha2 | -0.53289 | 0.002491 | 0.035062 |
| Eln | -0.4278 | 0.002502 | 0.035179 |
| LOC301124 | 0.735916 | 0.002511 | 0.035263 |
| Smtnl2 | 0.476467 | 0.002519 | 0.035342 |
| Ptbp2 | -0.58324 | 0.002531 | 0.035473 |
| Arl6ip5 | 0.487158 | 0.002544 | 0.035631 |
| Gpr137 | 0.39502 | 0.00257 | 0.035952 |
| AABR07054593.1 | 0.563994 | 0.002573 | 0.035958 |
| Dnajc22 | 0.484504 | 0.002576 | 0.035959 |
| Tra2a | -0.51966 | 0.002596 | 0.0362 |
| Fhl3 | 0.442019 | 0.002601 | 0.036235 |
| Pcnt | -0.82083 | 0.002608 | 0.036269 |
| LOC100909732 | -0.93949 | 0.002607 | 0.036269 |
| Dis3 | -0.37929 | 0.002619 | 0.036387 |
| Bag1 | 0.552468 | 0.002622 | 0.036389 |
| Riok3 | 0.284684 | 0.00263 | 0.036456 |
| Nav2 | -0.71331 | 0.002638 | 0.036529 |
| Ptpmt1 | 0.514386 | 0.002656 | 0.036699 |
| Mybl1 | -0.65917 | 0.002659 | 0.036699 |
| Nucks1 | -0.29842 | 0.002658 | 0.036699 |
| Tpcn1 | 0.286688 | 0.00266 | 0.036699 |
| Rabepk | 0.48482 | 0.002664 | 0.036708 |
| Krt8 | 0.394377 | 0.00267 | 0.036756 |
| Rps18 | 0.755542 | 0.002684 | 0.036909 |
| Fancc | -0.76166 | 0.00269 | 0.036957 |
| Churc1 | 0.550293 | 0.002705 | 0.037053 |
| Slc25a39 | 0.493936 | 0.002704 | 0.037053 |
| AABR07058884.2 | -0.76443 | 0.002704 | 0.037053 |
| Prpf31 | -0.31009 | 0.002711 | 0.037108 |
| Agpat3 | 0.438586 | 0.002719 | 0.037115 |
| Myd88 | 0.420707 | 0.002721 | 0.037115 |
| Pnkp | 0.507903 | 0.002724 | 0.037115 |
| Krtcap2 | 0.549317 | 0.002725 | 0.037115 |
| Als2 | -0.4938 | 0.002722 | 0.037115 |
| Cdc73 | -0.44396 | 0.002768 | 0.037571 |
| Atr | -0.37364 | 0.002769 | 0.037571 |
| Bag3 | 0.344866 | 0.002772 | 0.037571 |
| Dpy19l3 | -0.58062 | 0.00277 | 0.037571 |
| Dctn2 | 0.460176 | 0.002768 | 0.037571 |
| Phospho2 | 0.384792 | 0.00278 | 0.037634 |
| Baz1a | -0.57014 | 0.002788 | 0.037714 |
| Tmem59 | 0.359202 | 0.00281 | 0.037941 |
| Myl6b | -0.69698 | 0.002808 | 0.037941 |
| LOC100359539 | -0.42615 | 0.002825 | 0.038067 |
| Atp6v1d | 0.486777 | 0.002824 | 0.038067 |
| Cdca4 | -0.32043 | 0.00285 | 0.038364 |
| N4bp3 | -1.25719 | 0.00286 | 0.038457 |
| Amz2 | 0.414629 | 0.002866 | 0.038505 |
| Rbmx | -0.35815 | 0.002875 | 0.038595 |
| Cib1 | 0.566654 | 0.002881 | 0.038627 |
| Raph1 | -0.67485 | 0.002885 | 0.038647 |
| Ung | -0.52071 | 0.002909 | 0.038925 |
| Ints2 | -0.5139 | 0.002911 | 0.038925 |
| Tmem38a | 0.364668 | 0.002923 | 0.039049 |
| Dtd2 | 0.545568 | 0.00293 | 0.039076 |
| Zfp382 | -0.77652 | 0.002931 | 0.039076 |
| Rangrf | 0.769758 | 0.002942 | 0.039188 |
| Timm10b | 0.663137 | 0.002951 | 0.039268 |
| RGD1310769 | 0.451327 | 0.002957 | 0.039309 |
| Ccdc14 | -0.52575 | 0.002997 | 0.039773 |
| Cisd2 | -0.36503 | 0.002996 | 0.039773 |
| Tmem14a | 0.6899 | 0.003001 | 0.039786 |
| Casp12 | 0.416442 | 0.003007 | 0.039824 |
| Tmem18 | 0.38947 | 0.003034 | 0.04011 |
| Lsm8 | -0.42379 | 0.003034 | 0.04011 |
| Tbc1d8b | -0.50064 | 0.003043 | 0.040187 |
| Ifngr1 | 0.359156 | 0.003049 | 0.040202 |
| LOC100361993 | 0.576367 | 0.00305 | 0.040202 |
| Rad18 | -0.55618 | 0.003056 | 0.040241 |
| Cald1 | -0.36427 | 0.003063 | 0.040296 |
| Irs2 | -0.49212 | 0.003066 | 0.040296 |
| Gpm6b | 0.370533 | 0.003077 | 0.040355 |
| Mcph1 | -0.52949 | 0.003079 | 0.040355 |
| Rwdd1 | 0.403542 | 0.003076 | 0.040355 |
| AABR07056633.1 | -0.8317 | 0.003085 | 0.040396 |
| B4galt2 | 0.345443 | 0.003107 | 0.040649 |
| Mafk | -0.40915 | 0.003138 | 0.041006 |
| Ptpn21 | -0.60666 | 0.003167 | 0.041358 |
| Hexb | 0.304464 | 0.003197 | 0.041699 |
| Bckdha | 0.554223 | 0.003202 | 0.041734 |
| Tef | 0.329504 | 0.003209 | 0.041787 |
| Nrip2 | 2.024301 | 0.003223 | 0.041885 |
| Tmco1 | 0.396467 | 0.003221 | 0.041885 |
| Dgcr8 | -0.39648 | 0.003235 | 0.041998 |
| Idi1 | 0.325946 | 0.003244 | 0.042007 |
| LOC361985 | 0.566974 | 0.003244 | 0.042007 |
| Ei24 | 0.362851 | 0.003239 | 0.042007 |
| Grn | 0.24038 | 0.003251 | 0.04205 |
| RGD1303003 | 0.495036 | 0.00326 | 0.042121 |
| Nit1 | 0.394146 | 0.003262 | 0.042121 |
| Zcchc8 | -0.57272 | 0.003276 | 0.042178 |
| Vamp4 | 0.412866 | 0.003274 | 0.042178 |
| Atp6v0e1 | 0.455892 | 0.003281 | 0.042178 |
| Nipal2 | 0.581652 | 0.003285 | 0.042178 |
| Elf4 | -0.39098 | 0.003275 | 0.042178 |
| Pcyt2 | 0.33781 | 0.003283 | 0.042178 |
| Tchp | -0.33531 | 0.003288 | 0.042178 |
| Haus3 | -0.60872 | 0.003298 | 0.042266 |
| Traf3ip1 | -0.67028 | 0.003301 | 0.042269 |
| Eri2 | -0.41067 | 0.003305 | 0.042287 |
| Tex261 | 0.550294 | 0.003314 | 0.042353 |
| Grip2 | -1.196 | 0.003328 | 0.042395 |
| Rtkn2 | -0.64953 | 0.003321 | 0.042395 |
| Timm8b | 0.473071 | 0.003325 | 0.042395 |
| Cebpa | 0.832789 | 0.003329 | 0.042395 |
| Setd1a | -0.58464 | 0.003347 | 0.042585 |
| LOC100362620 | -0.33789 | 0.003361 | 0.042624 |
| Bicd2 | -0.52616 | 0.003358 | 0.042624 |
| Katnal1 | 0.524922 | 0.003356 | 0.042624 |
| Lmbrd2 | -0.58618 | 0.003362 | 0.042624 |
| RGD1560010 | -0.46198 | 0.003389 | 0.042733 |
| Saraf | 0.309997 | 0.003383 | 0.042733 |
| Rnaset2 | 0.54105 | 0.003389 | 0.042733 |
| Las1l | -0.43963 | 0.003387 | 0.042733 |
| Il17rc | 0.453448 | 0.00338 | 0.042733 |
| Ube2m | 0.480906 | 0.003376 | 0.042733 |
| Pcdhb17 | -1.14608 | 0.003412 | 0.042828 |
| Opn3 | 0.974022 | 0.003412 | 0.042828 |
| Msantd3 | 0.374607 | 0.003408 | 0.042828 |
| Mutyh | -0.75366 | 0.003405 | 0.042828 |
| Ormdl2 | 0.522736 | 0.003408 | 0.042828 |
| Hnrnpu | -0.50864 | 0.00342 | 0.042888 |
| RGD1310712 | -0.72343 | 0.003424 | 0.042902 |
| Osbpl3 | -0.47812 | 0.003443 | 0.043093 |
| Setd1b | -0.70519 | 0.003458 | 0.043241 |
| Atp11c | -0.50464 | 0.003489 | 0.043564 |
| Prdx3 | 0.450686 | 0.003499 | 0.043564 |
| Slc25a12 | 0.35997 | 0.003499 | 0.043564 |
| AABR07027388.1 | 0.563213 | 0.003494 | 0.043564 |
| Rpn1 | 0.334303 | 0.003493 | 0.043564 |
| Srsf9 | 0.307689 | 0.003511 | 0.043639 |
| Uchl5 | -0.43625 | 0.00351 | 0.043639 |
| Galnt16 | 0.301003 | 0.003517 | 0.043664 |
| Sec24a | -0.61603 | 0.00352 | 0.043665 |
| LOC100911516 | 0.752484 | 0.003525 | 0.043693 |
| Rrp1b | -0.2978 | 0.003539 | 0.043767 |
| Tceal8 | -0.24855 | 0.003537 | 0.043767 |
| Rnf187 | 0.423381 | 0.003541 | 0.043767 |
| Entpd7 | -0.48501 | 0.003566 | 0.043965 |
| Nadk | 0.297739 | 0.003564 | 0.043965 |
| Nfatc2ip | -0.51039 | 0.003565 | 0.043965 |
| Ddx39b | -0.28999 | 0.003588 | 0.044154 |
| Sdf2 | 0.424403 | 0.003586 | 0.044154 |
| Sphk1 | 0.429476 | 0.003597 | 0.044203 |
| Uqcrq | 0.582158 | 0.003598 | 0.044203 |
| Rnpepl1 | 0.417517 | 0.003608 | 0.044288 |
| Cers5 | 0.326044 | 0.003615 | 0.044335 |
| LOC100909750 | -0.38609 | 0.003626 | 0.044427 |
| Serf1 | 0.945492 | 0.003629 | 0.04443 |
| Syn1 | 0.534482 | 0.003649 | 0.044634 |
| Rab13 | 0.459303 | 0.003664 | 0.044771 |
| Myo19 | -0.47995 | 0.003677 | 0.044855 |
| Rbm15 | -0.50829 | 0.003675 | 0.044855 |
| Nfrkb | -0.40665 | 0.003681 | 0.044855 |
| Rabac1 | 0.532883 | 0.003689 | 0.044855 |
| Shisa5 | 0.361124 | 0.00369 | 0.044855 |
| LOC100359574 | 0.959629 | 0.003686 | 0.044855 |
| Nr4a2 | -0.69441 | 0.003701 | 0.044871 |
| Lrrfip1 | -0.58454 | 0.0037 | 0.044871 |
| Bcap31 | 0.487958 | 0.003697 | 0.044871 |
| Slfn3 | -1.09326 | 0.003704 | 0.044873 |
| Rad51c | -0.52012 | 0.003731 | 0.04512 |
| Higd2a | 0.415165 | 0.00373 | 0.04512 |
| Smpd1 | 0.253212 | 0.003735 | 0.045125 |
| AC095263.1 | 0.686648 | 0.003752 | 0.045289 |
| Xpo4 | -0.45219 | 0.003766 | 0.045362 |
| Eif3i | 0.605565 | 0.003766 | 0.045362 |
| Ldlrad3 | -0.5789 | 0.003767 | 0.045362 |
| Tcf4 | -0.54417 | 0.003773 | 0.045392 |
| Tgif2-ps1 | -1.81969 | 0.003782 | 0.045426 |
| LOC100362384 | 1.052201 | 0.003782 | 0.045426 |
| Micu1 | 0.367197 | 0.003786 | 0.045426 |
| Uspl1 | -0.32458 | 0.003808 | 0.045557 |
| Fibp | 0.505504 | 0.003813 | 0.045557 |
| Prtfdc1 | 0.587327 | 0.00381 | 0.045557 |
| Srrt | -0.3279 | 0.003816 | 0.045557 |
| Mapk8ip1 | 0.476355 | 0.003816 | 0.045557 |
| BC055324 | -0.54481 | 0.003804 | 0.045557 |
| Nupl1 | -0.38638 | 0.003839 | 0.045788 |
| Sarm1 | 0.656674 | 0.003843 | 0.045796 |
| Arid5b | -0.5376 | 0.003853 | 0.045882 |
| S1pr3 | 0.254463 | 0.003898 | 0.04637 |
| Ccdc82 | -0.47315 | 0.003906 | 0.046399 |
| Cd276 | 0.267924 | 0.003907 | 0.046399 |
| Shcbp1 | -0.49584 | 0.003919 | 0.0465 |
| Mxra8 | 0.278941 | 0.00393 | 0.046596 |
| Klhl9 | -0.49083 | 0.003945 | 0.046736 |
| AABR07000550.1 | 0.970896 | 0.003953 | 0.046791 |
| Pgls | 0.617139 | 0.003961 | 0.046839 |
| LOC100911224 | -0.45637 | 0.003967 | 0.046875 |
| Porcn | 0.331069 | 0.003981 | 0.047003 |
| Pla2g4d | 2.899066 | 0.003985 | 0.047005 |
| Trip13 | -0.46234 | 0.004003 | 0.047178 |
| Usp11 | 0.358633 | 0.004009 | 0.04721 |
| Cox7a2l | 0.500609 | 0.004021 | 0.047306 |
| LOC100360260 | -0.2709 | 0.004024 | 0.047307 |
| Lamtor2 | 0.394842 | 0.004048 | 0.047543 |
| Yif1a | 0.544212 | 0.004061 | 0.047657 |
| Rn60_16_0006.1 | -1.66091 | 0.004065 | 0.047672 |
| AABR07044711.1 | 0.54484 | 0.004071 | 0.047701 |
| Hilpda | 1.316863 | 0.004102 | 0.047981 |
| Eif4e3 | 0.68829 | 0.0041 | 0.047981 |
| Slc4a4 | -0.97986 | 0.004119 | 0.048144 |
| Eif3f | 0.551666 | 0.004134 | 0.048271 |
| Lrrc73 | 1.01721 | 0.004146 | 0.048372 |
| Tnfrsf1b | 0.886274 | 0.004178 | 0.048707 |
| Lemd2 | 0.330214 | 0.004213 | 0.049074 |
| Tspan9 | -0.29809 | 0.004223 | 0.049147 |
| Popdc2 | -0.79814 | 0.004231 | 0.0492 |
| Mien1 | 0.706714 | 0.004275 | 0.049426 |
| Ipo9 | -0.27015 | 0.004262 | 0.049426 |
| Pdp2 | -0.36117 | 0.00427 | 0.049426 |
| Gpx4 | 0.637386 | 0.004261 | 0.049426 |
| Gtf2h5 | 0.513586 | 0.004267 | 0.049426 |
| Tmem126b | 0.497094 | 0.004274 | 0.049426 |
| Myrf | -0.34007 | 0.004266 | 0.049426 |
| Kank4 | -0.70608 | 0.004292 | 0.049542 |
| Bod1l1 | -0.79646 | 0.004293 | 0.049542 |
| Dlc1 | -0.61312 | 0.004301 | 0.049595 |
| Nbl1 | 0.327781 | 0.004324 | 0.049827 |
| Cited1 | 0.65266 | 0.004342 | 0.049905 |
| Lpcat4 | 0.408838 | 0.004336 | 0.049905 |
| Ica1 | 0.651463 | 0.00434 | 0.049905 |
